# The Human Papillomavirus E6 Oncogene Represses a Cell Adhesion Pathway and Disrupts Focal Adhesion through Degradation of TAp63β upon Transformation

**DOI:** 10.1371/journal.ppat.1002256

**Published:** 2011-09-29

**Authors:** Youcef Ben Khalifa, Sébastien Teissier, Meng-Kwang Marcus Tan, Quang Tien Phan, Mathieu Daynac, Wei Qi Wong, Françoise Thierry

**Affiliations:** Institute of Medical Biology, A*STAR, Singapore; University of Wisconsin, United States of America

## Abstract

Cervical carcinomas result from cellular transformation by the human papillomavirus (HPV) E6 and E7 oncogenes which are constitutively expressed in cancer cells. The E6 oncogene degrades p53 thereby modulating a large set of p53 target genes as shown previously in the cervical carcinoma cell line HeLa. Here we show that the TAp63β isoform of the p63 transcription factor is also a target of E6. The *p63* gene plays an essential role in skin homeostasis and is expressed as at least six isoforms. One of these isoforms, ΔNp63α, has been found overexpressed in squamous cell carcinomas and is shown here to be constitutively expressed in Caski cells associated with HPV16. We therefore explored the role of p63 in these cells by performing microarray analyses after repression of endogenous E6/E7 expression. Upon repression of the oncogenes, a large set of p53 target genes was found activated together with many p63 target genes related to cell adhesion. However, through siRNA silencing and ectopic expression of various p63 isoforms we demonstrated that TAp63β is involved in activation of this cell adhesion pathway instead of the constitutively expressed ΔNp63α and β. Furthermore, we showed in cotransfection experiments, combined with E6AP siRNA silencing, that E6 induces an accelerated degradation of TAp63β although not through the E6AP ubiquitin ligase used for degradation of p53. Repression of E6 transcription also induces stabilization of endogenous TAp63β in cervical carcinoma cells that lead to an increased concentration of focal adhesions at the cell surface. Consequently, TAp63β is the only p63 isoform suppressed by E6 in cervical carcinoma as demonstrated previously for p53. Down-modulation of focal adhesions through disruption of TAp63β therefore appears as a novel E6-dependent pathway in transformation. These findings identify a major physiological role for TAp63β in anchorage independent growth that might represent a new critical pathway in human carcinogenesis.

## Author Summary

High-risk human papillomavirus infection can cause cancer of the uterine cervix. The viral proteins leading to transformation of the infected keratinocytes are the E6 and E7 oncogenes which interact with and induce degradation of the cell cycle regulators p53 and pRB. In cervical carcinoma cells, repression of E6/E7 stabilizes the p53 transcription factor leading to activation of a large group of cellular p53 target genes. Here we show that repression of E6/E7 also induces transcriptional activation of an additional large set of genes involved in cell adhesion including previously described p63 target genes. Indeed, we further demonstrated that these p63 target genes are activated by TAp63β and not by p53 or by the ΔNp63α or β isoforms, even though these transcription factors are also expressed in these cells. In cervical carcinoma cells, E6 expression therefore leads to TAp63β degradation thereby allowing anchorage independent growth. Our work describes a new E6-dependent transformation pathway in HPV-associated carcinogenesis. TAp63β inhibition may also represent a common pathway to activate anchorage independent growth in cancers.

## Introduction

Infection of the anogenital mucosal epithelium with high risk Human Papilloma Virus (HPV) is linked to 99% of cervical carcinomas [1]. Cell lines derived from these cervical carcinomas remain associated with HPV and contain part of the viral genome integrated in the cellular genome. However, not all viral genes are retained in this integration; the E6 and E7 oncogenes remain, while the open reading frames encoding viral proteins E1 and E2, necessary for viral DNA replication, are disrupted [2], [3]. We have previously used the HPV18-associated HeLa cell line to study transcriptional modulation of viral and cellular genes following repression of the E6 and E7 oncogenes, and found that a large number of cellular genes were in fact modulated via E6 and E7 [4], [5]. Of particular interest was the discovery that genes targeted by either p53 or E2F were respectively activated or repressed through repression of E6 and E7 [4].

We now wish to develop and extend these findings. In particular we are interested in the potential effect of HPV E6 and E7 on other less well defined members of the p53 family. The p73 and p63 transcription factors are more recently discovered p53 family members, and although they share structural homology with p53 and are able to interact with similar DNA binding motifs, they modulate different regulatory pathways [6]–[9]. While p53 is a tumor suppressor and does not obviously participate in embryonic development, p63 and p73 on the contrary are strongly linked to embryonic development in mice [10], [11].

The critical developmental role of p63 is illustrated in null mice, which do not survive beyond few days after birth and exhibit limb truncation and a striking absence of skin [10], [11]. Human genetic syndromes with comparable phenotypes have also been linked to p63, including EEC syndrome (Ectrodactyly Ectodermal dysplasia Clefting), ADULT (Acro-Dermato-Ungual-Lacrimal-Tooth malformations), LMS (Limb-Mammary syndrome), Hay Wells syndrome, SHFM (Split-hand/foot malformations), and Rapp-Hodgkin Syndrome, reviewed in [12]. These findings of a crucial role of p63 in skin development were supported by its specific expression pattern in epithelia [13]. There is also evidence that p63 may play a role in cancer since it has been shown to be overexpressed in squamous cell carcinomas [14]. In addition, the p63 proteins are transcription factors that modulate a large cluster of genes, which differ from p53 target genes [15]–[19]. One specific cluster of p63 target genes is related to cellular adhesion and contains either cell: matrix or cell: cell adhesion genes such as integrins, and components of the ECM, desmosomes or tight junctions.

Interpreting the potential role of p63 in cancer is complicated by the existence of six isoforms of the transcription factor. The p63 gene contains two promoters driving the production of distinct proteins, one with an N-terminal transactivation domain (TAp63) and one without (ΔNp63). Moreover, each of these two proteins may be expressed as α, β and γ isoforms, exhibiting particular expression patterns and characteristics. For example, ΔNp63α is abundantly expressed in proliferating keratinocytes of the epithelial basal layers, while the TA isoforms are not detectable in skin [20]. The ΔNp63α isoform is also increased in squamous cell carcinomas [13], thus supporting its proliferative functions. In contrast, TAp63 has recently been described as preventing premature aging by promoting adult stem cell maintenance in mice, linking its activities to DNA repair and wound healing rather than to differentiation [21]. Although this isoform of p63 is able to functionally interact with ΔNp63, p73 and p53, it also displays a specific role as transcriptional modulator of its own target genes such as the kinase inhibitor p57^kip2^ [21]. Roles of the various p63 isoforms as well as p53 and p73 in skin proliferation and differentiation are not yet fully understood. Additional complexity comes from the fact that they are able to compensate each other to some extent, as demonstrated for example by the increase of p63 expression in the skin of the p53 -/- mice [22]. They are also able to regulate each other, although these cross regulations are not yet fully understood and involve transcriptional as well as post transcriptional mechanisms [23].

Here we show that repression of the E6/E7 oncogenes induces transcriptional activation of a large set of potential p63 target genes in the HPV16-associated cervical carcinoma Caski cell line. We demonstrate, through siRNA silencing and direct over-expression of various p63 isoforms, that some of these genes are indeed p63 targets, mostly responsive to TAp63β and not to the constitutively expressed ΔNp63α and β isoforms. Furthermore we showed that the stability of the TAp63β isoform is down-modulated by E6, thus functionally linking repression of p63 target genes to expression of the E6 oncogene in cervical carcinoma cells. Over-expression of TAp63β or repression of E6, both induce increase in focal adhesion in cervical carcinoma cell lines. These data emphasize a new role of the TAp63β isoform in activating cell adhesion in epithelial cells and of the E6 oncogene in down-modulating this pathway in cervical cancer.

## Results

### Expression of p63 isoforms in cervical carcinoma and keratinocyte cell lines

**Figure 1:**
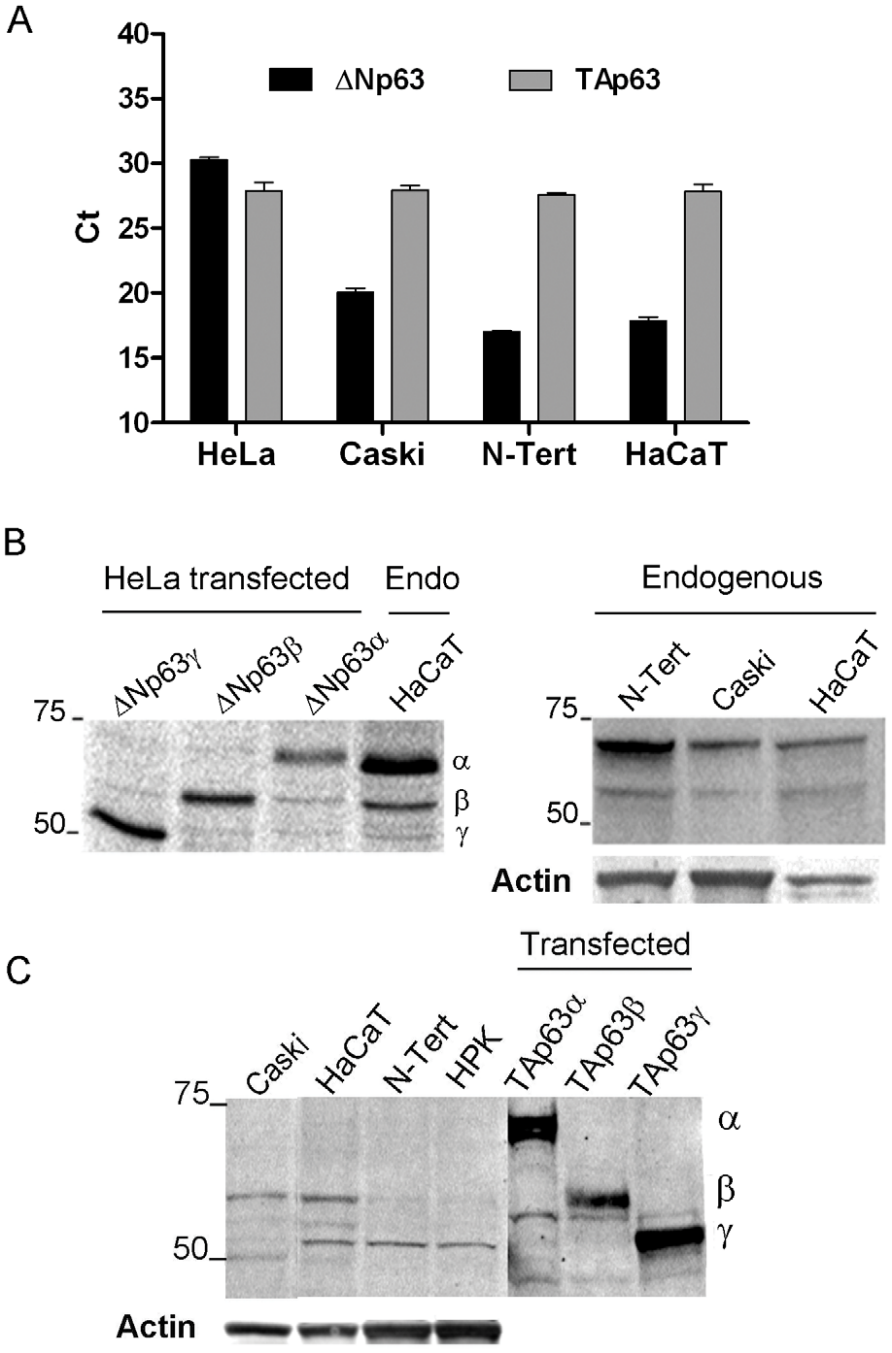
Levels of expression of endogenous p63 isoforms in four keratinocyte cell lines. (A) Real-Time PCR analyses of endogenous ΔNp63 and TAp63 isoforms performed in two cervical carcinoma cell lines, HeLa and Caski associated with HPV18 and HPV16 respectively, and two immortalized keratinocyte cell lines not associated with HPV, N-Tert and HaCaT. Values given are Ct (Cycling threshold) with higher Ct corresponding to lower mRNA levels; data are represented as mean ± standard deviation (SD). Internal controls were mRNA levels of housekeeping genes *HDAC*, *GADPH* and 18S rRNA. (B) Western blot analyses of endogenous levels of ΔNp63 isoforms in Caski, HaCaT and N-Tert cells compared to HeLa cells transfected with the expression vectors of the different ΔNp63 isoforms (α, β and γ) detected using a pan-p63 antibody (4A4 Pharmingen). Quantification of the western blots indicated that N-TERT and HaCaT express 1.5 and 1.9-fold more ΔNp63α than Caski. (C) Western blot analyses of endogenous levels of TAp63 isoforms in Caski, HaCaT, N-Tert, and human primary keratinocytes (HPK) using a purified specific TAp63 antibody (Biolegend Poly 6189) compared to HeLa cells transfected with the vectors expressing the TAp63 isoforms α, β and γ. Quantification of the western blots indicated that HaCaT express 1.3-fold more TAp63β than Caski while the γ isoform is expressed 2.3-fold more in HaCaT and N-Tert and 1.6-fold more in HPK than in Caski.

We wished to understand whether a p63-mediated adhesion pathway might be important in the HPV16-associated cervical carcinoma Caski cell line and how these cells express p63 compared to HeLa cells and non transformed human keratinocyte cell lines. Levels of mRNA for the ΔN and TA isoforms of p63 were measured by Real Time PCR in HeLa and Caski cancer cell lines, compared to the non HPV-associated keratinocyte cell lines HaCaT and N-Tert. Low levels of transcripts (around 28 ct) of the TAp63 isoforms were detected in all four cell lines, while transcript levels of the ΔNp63 isoforms varied from very low in HeLa cells (30 ct) to markedly higher levels in the HaCaT, N-Tert (around 18 ct) and Caski cells (around 20 ct) (Figure 1A). In HeLa cells, no endogenous p63 isoforms were detected at the protein level, as expected from the low levels of transcription (data not shown). We took advantage of this and used HeLa cells transfected with expression vectors of the different p63 isoforms as a comparison to study endogenous p63 protein levels in the other cell lines. Only 2 isoforms of ΔNp63 (α and β) were consistently detected in N-Tert, HaCaT and Caski cell extracts with an expression level of ΔNp63α between 1,5 and 2 times higher in non HPV-associated keratinocytes than in Caski (Figure 1B). Under similar conditions, TAp63 isoforms were not detectable in any of these cell extracts. However, low levels of TAp63 were detected with the use of a purified antibody against the TA domain of p63. Caski cells express mainly the TAp63β isoform while HaCaT cells express both β and γ isoforms and N-Tert and primary keratinocytes mainly the γ isoform with very low levels of the β isoform (Figure 1C).

### Repression of HPV16 E6/E7 transcription modulates a p63 pathway in Caski cells

**Figure 2:**
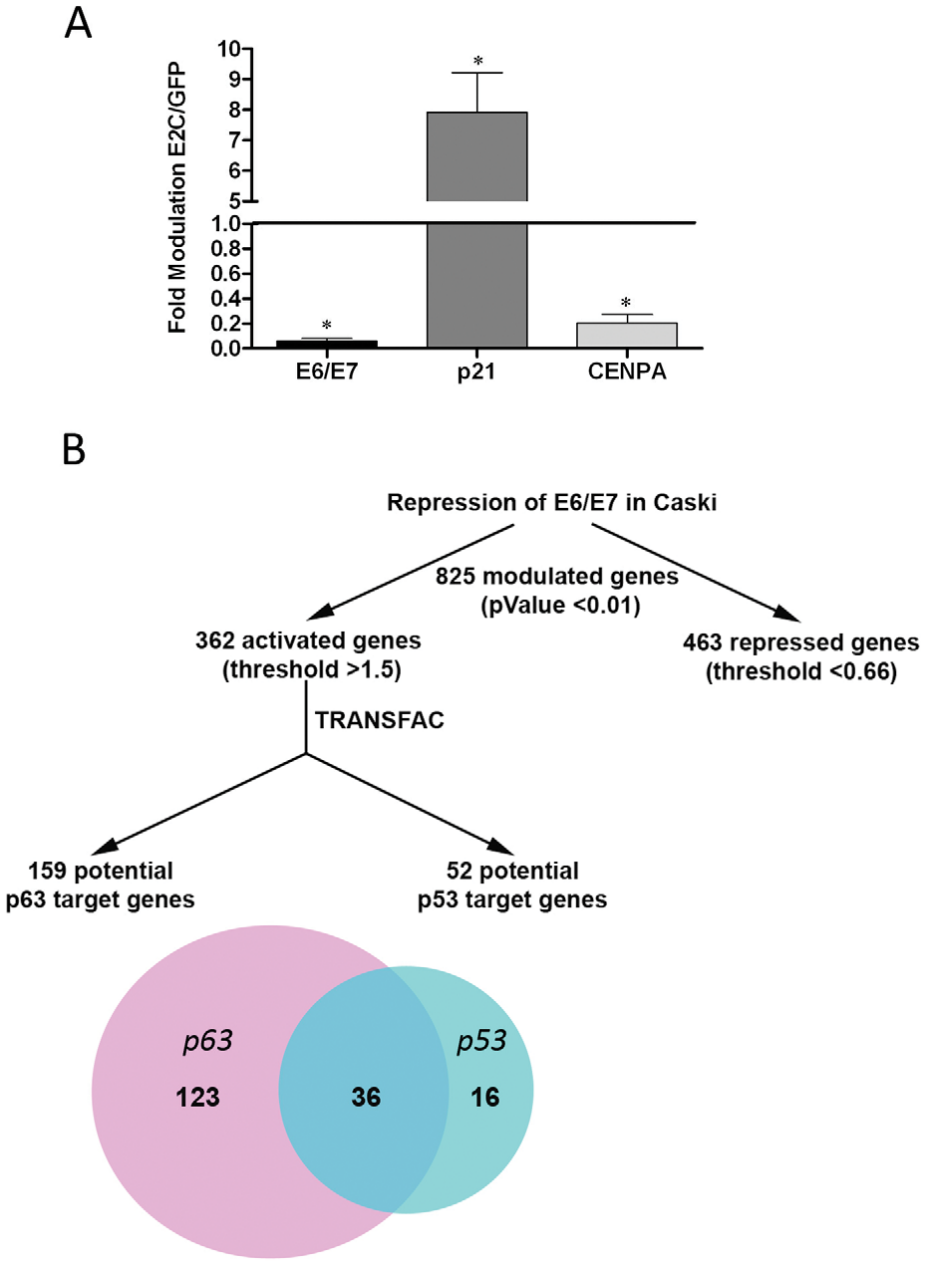
Transcriptome analyses in Caski cells after E2C-induced transcriptional repression of E6 and E7. (A) Modulation of E6/E7, *p21* (E6/p53 pathway) and *cenpa* (E7/E2F pathway) mRNA levels by Real-Time PCR in Caski cells infected by adenoviruses expressing GFP-E2C or GFP in three independent experiments. Values given are the ratio of mRNA levels in E2C compared to GFP infected cells (mean ± SD, *p < 0.05). (B) Schematic representation of the modulation of cellular genes found in microarray experiments performed in Caski cells. Data were analyzed using Partek Genomics Suite software which revealed a total of 825 modulated genes including 463 repressed and 362 activated genes. Using TRANSFAC Professional software (Biobase), the list of activated genes was compared to p53 and p63 Chip on ChIP data.

In a previous study we described the modulation of putative p63 target genes in HeLa cells following HPV18 E6/E7 repression [5]. This occurred in the absence of detectable endogenous p63 expression; therefore we asked whether the range of p63 target genes modulated might be greater in Caski cells, which express significant levels of endogenous p63. A truncated form of the HPV protein E2 efficiently represses E6/E7 transcription in HeLa cells [4], and thus may be exploited as a reagent to study the effects of their inhibition. We used Affymetrix microarrays to study gene modulation in Caski cells infected by recombinant adenoviruses expressing GFP or the GFP-E2 protein truncated of its transactivation domain (E2C). As revealed by Real Time PCR, E2C repressed E6/E7 transcription by more than 90% under conditions where 100% of Caski cells were infected (Figure 2A). We also verified the activation of the endogenous p53 target gene *p21* (8-fold) and the repression of the cell cycle gene *cenpa* (5-fold), that have previously been shown to be modulated by repression of E6/E7 in cervical carcinoma cells (Figure 2A) [5]. Gene expression analysis in E6/E7-repressed Caski cells revealed modulation of 825 genes: 463 repressed genes, including mitotic genes and E2F target genes (data not shown), and 362 genes whose activation was increased by more than 1.5-fold, with a p-Value less than 0.01 (data not shown). Using the TRANSFAC Professional data base (Biobase) we assigned 52 of these activated genes to the p53 pathway (Table S1) while 159 could be assigned to the p63 pathway (Table S2), with 36 genes overlapping with p53 (Figure 2B). In all, 123 exclusive p63 putative target genes were regulated following HPV16 E6/E7 repression. Of these, approximately 20% have previously been allocated to adhesion pathways [5], [15] (Table S3 in Text S1).

To confirm up-regulation of an adhesion pathway upon repression of E6 and E7 transcription, Caski cells infected with the adenovirus encoding GFP-E2C compared to the GFP control were subjected to gene expression analyses using a Human extracellular matrix and adhesion molecules PCR array (SABiosciences). Repression of E6/E7 transcription by E2C induced a strong response in genes involved in cellular adhesion in Caski cells, with 34% of the genes present in the PCR-arrays activated between 1.8 and 7-fold and 6% of genes repressed (Figure S1A in Text S1). Silencing E6/E7 with siRNA was also efficient, activating 53% of the PCR-arrays genes while only 3% were repressed (Figure S1B in Text S1). Among the genes contained within these arrays, 18 are known p63 targets. Half of these genes were activated in both conditions of E6/E7 repression.

### Modulation of putative p63 target genes by E6/E7 is independent of both p53 and ΔNp63 isoforms

**Figure 3:**
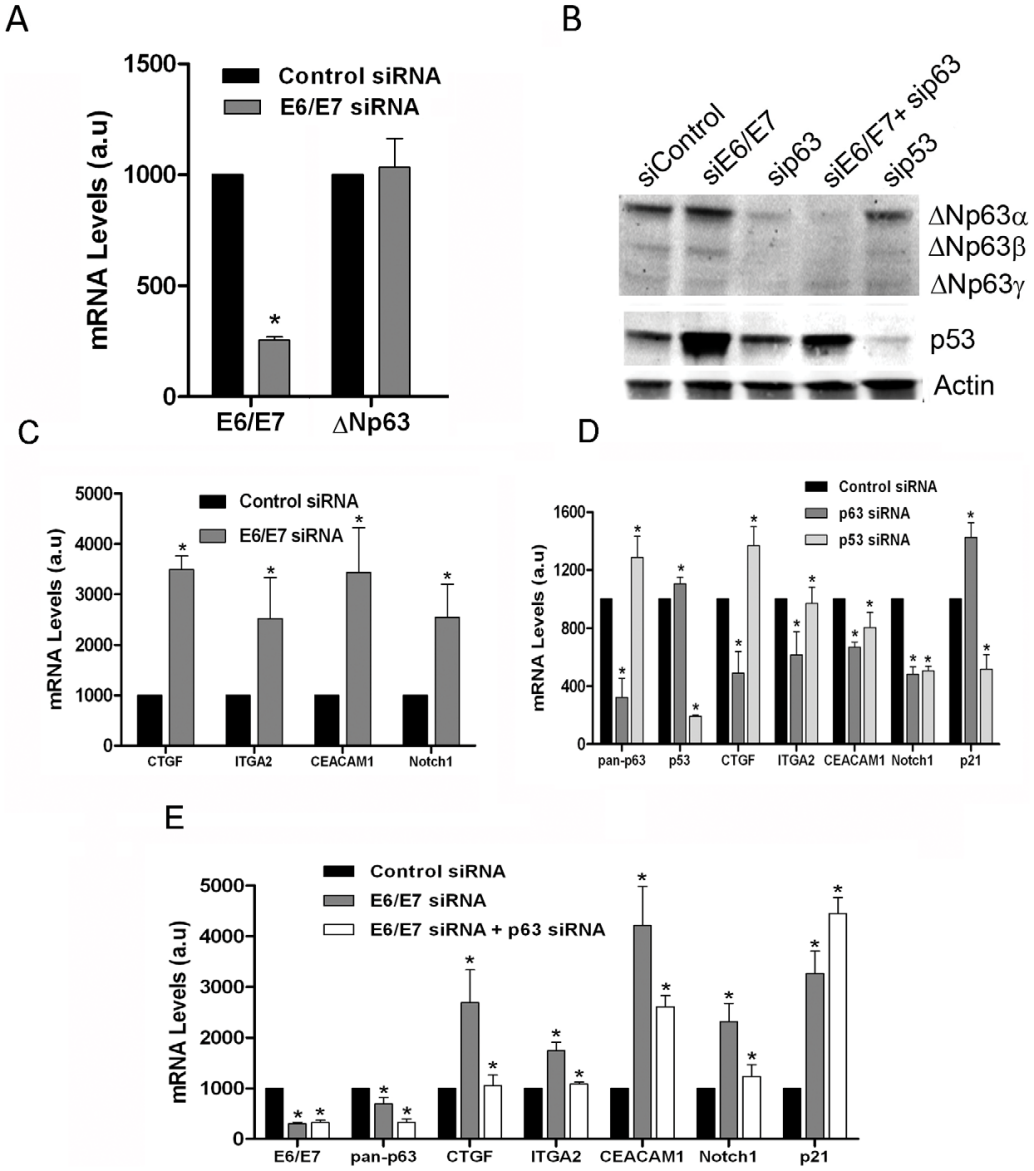
Modulation of a set of adhesion genes in Caski through E6/E7 silencing is dependent on p63. (A) Real-Time PCR of the E6/E7 oncogenes and ΔNp63 performed in Caski cells expressing E6/E7 siRNA compared with siRNA control where mRNA levels are given in arbitrary units (a.u) with the control siRNA set up to 1000 (mean ± SD, *p < 0.05). (B) Western blot analyses of endogenous ΔNp63 isoforms and p53 in Caski expressing different siRNA showing silencing of all ΔNp63 isoforms (α, β and γ) by the p63 siRNA (40 and 50% in the absence or presence of siE6/E7) and 80% silencing of p53 by the p53 siRNA. A relative stabilization of p53 upon silencing of E6/E7 is also visible. (C) Modulation of mRNA levels of the set of adhesion genes from (Table S3 in Text S1), following silencing of E6/E7. (D) Modulation of mRNA levels of the same set of genes following silencing of p63 or p53. (E) Concomitant silencing of p63 and E6/E7 partially restored the basal levels of the adhesion genes but not of the p53 target gene, *p21*. mRNA levels were obtained by RT-PCR and calculated as in panel A (mean ± SD, *p < 0.05).

As siRNA was also effective in repressing E6/E7 transcription in Caski cells (Figure S1B in Text S1), we used this technique to further appreciate the effects of the HPV oncogenes on the p63 pathway. We used E6/E7 siRNA to silence mRNA expression of both viral oncogenes together, since they are expressed from a single polycistronic mRNA and cannot be silenced individually [5]. Silencing of E6/E7 by about 60% did not affect levels of endogenous ΔNp63 mRNA (Figure 3A). This was confirmed by western blots, using a pan-p63 antibody (4A4), with pan-p63 siRNA (which silenced all p63 isoforms) and p53 siRNA as positive and negative controls respectively (Figure 3B upper panel). The p63 siRNA decreased p63 protein expression by about 50% while the p53 siRNA decreased p53 expression by 80% as quantified from the western blots shown in Figure 3B. In contrast, E6/E7 siRNA induced stabilization of the p53 protein, which was stabilized even in the presence of cotransfected p63 siRNA (Figure 3B lower panel). We then selected a group of the putative p63 target genes identified as activated in the microarrays from the list of genes given in (Table S3 in Text S1) and assessed their levels of transcription following siRNA silencing of E6/E7. As expected, these genes were activated between 3 and 4-fold following silencing of E6 and E7 transcription, thus confirming the microarray data (Figure 3C). Interestingly however, these experiments lead to the conclusion that HPV16 E6/E7 cause down regulation of putative p63 target genes without modulating ΔNp63.

The fact that E6/E7 silencing did not affect ΔNp63 expression while it stabilizes p53 (Figure 3B) led us to ask whether the cellular target genes were indeed being modulated by p63. Direct silencing of p63 by a pan-p63 siRNA induced up to 50% repression of the set of target genes, except for the p53 target gene *p21* (Figure 3D), while silencing p53 did not strongly influence their expression except in the case of *notch1* and *p21* (Figure 3D). We decided to confirm whether activation of these genes following E6/E7 silencing occurred through p63 by concomitantly transfecting the two siRNA. Cotransfection of Caski cells with the E6/E7 and p63 siRNA induced a reversion of the gene activation seen with the E6/E7 siRNA alone, thus indicating that these genes are indeed mainly controlled by p63 (Figure 3E). Activation of *p21* and both *notch* and *ceacam1* was either not or partially reversed by cotransfection of p63 siRNA, confirming that *p21* is mainly activated by p53 in Caski cells, while *notch1* and *ceacam1* are activated by both p63 and p53 as previously published for *notch1* [24], [25]. Therefore, although this panel of genes is indeed activated by p63, the ΔN isoforms constitutively expressed in Caski cells may not be directly involved in this regulation, since their expression is not modulated upon E6/E7 silencing (Figure 3A and 3B upper panel). As the p63 siRNA was designed to repress both ΔN and TA isoforms of p63, we therefore examined a possible role for the TAp63 and specifically of the beta isoform that is expressed in Caski cells (Figure 1C).

### The p63 target genes are directly activated by the TAp63β isoform

We first measured the effect of the six over-expressed p63 isoforms [26] or p53 on the transcription of previously described groups of endogenous p63 and p53 target genes in transfected HeLa cells [5]. The endogenous p63 target genes were activated by ectopic expression of the longer TA α and β isoforms and not by the shorter γ isoform (Figure S2A in Text S1). The ΔN isoforms were less active, although the β isoform did exhibit some level of activation (Figure S2B in Text S1). None of these genes were activated by p53, but *p21* was activated around 10-fold (Figure S2C in Text S1). While p53 did not activate p63 target genes, we found that the TAp63γ isoform efficiently activates p53 target genes including *p21* (Figure S2D in Text S1). These results demonstrated that a cross-activation of the p53 target genes by p63 can occur, although with marked preference for the shorter TAp63γ isoform, as previously reported [27].

**Figure 4:**
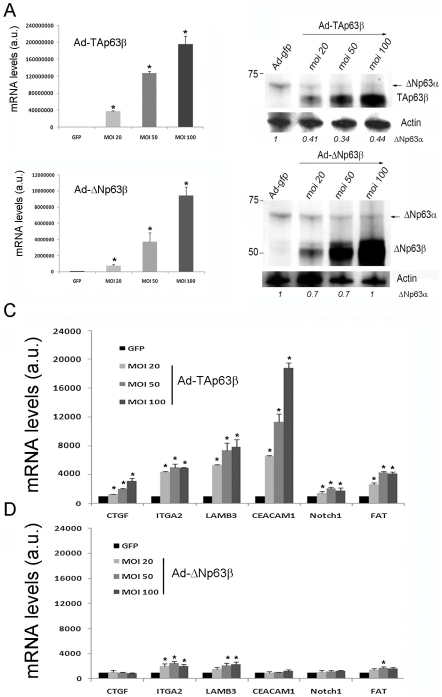
The adhesion genes are activated by TAp63β but not by ΔNp63β in Caski cells. (A) Real-time PCR performed in Caski cells infected with recombinant adenoviruses expressing GFP compared to TAp63β (upper panel) or ΔNp63β (lower panel) at increasing m.o.i. (20, 50 and 100). Levels of mRNA were given in arbitrary units (a.u) with the endogenous levels in GFP-infected cells set up at 1000. (B) Western Blots performed in infected Caski cells as described in panel A. The protein levels correlate with the m.o.i. and over expression of TAp63β and not ΔNp63β led to a decrease of the endogenous level of the ΔNp63α isoform as indicated by the quantification levels given below the western blot pictures. (C) Modulation of the mRNA levels of adhesion genes detected by RT-PCR in Caski cells infected with an increasing m.o.i. of the recombinant adenovirus expressing the TAp63β isoform compared to GFP. (D) Same experiments as in panel C with the adenovirus expressing the ΔNp63β isoform compared to adenovirus expressing GFP. Levels of mRNA were calculated as in A (mean ± SD, *p < 0.05). Data shown is representative of at least three independent experiments.

We then decided to confirm which p63 isoforms regulated the endogenous set of p63 target genes in Caski cells. We used recombinant adenoviruses expressing both α and β isoforms of TA and ΔNp63β that were previously found to be the most efficient in activating p63 target genes in transfected HeLa cells (Figure S2A and S2B in Text S1). Infection with an increasing multiplicity of infection (m.o.i.) of the recombinant adenovirus expressing the TAp63β isoform led to increased expression of the transcription factor (Figure 4A and 4B, upper panels), coupled with corresponding increases in mRNA expression of the endogenous set of genes (Figure 4C). Activation ranges from 2-fold for *notch1* to 20-fold for *ceacam1* with TAp63β (Figure 4C) while in contrast the ΔNp63β isoform (Figure 4A and 4B lower panels) did not induce significant changes in the basal mRNA levels of the p63 target genes, a maximum 2-fold change being seen for the *itga2* and *lamb3* gene expression (Figure 4D). The TAp63α long isoform was similarly inactive in this assay (Data not shown). Interestingly, we found that overexpression of the TAp63β isoform, but not of the ΔNp63β isoform, led to a decrease of about 3-fold of the endogenous constitutive level of the ΔNp63α isoform through an unknown mechanism (Figure 4B, upper panel). These results unambiguously establish that the set of genes modulated in our microarrays is composed of p63 target genes, likely activated most efficiently by the TAp63β isoform in Caski cells.

We then wanted to extend our finding to other cervical carcinoma cell lines and used SiHa also associated with HPV16. We first demonstrated that repression of E6/E7 transcription by E2C induced the same p63 target genes as in Caski cells as well as the p53 target gene *p21* (Figure S3A in Text S1). We also infected SiHa cells with the recombinant adenoviruses expressing TA and ΔN p63β isoforms using conditions where 100 % of the cells were infected (Figure S3B in Text S1). This showed that expression of TAp63β and not ΔNp63β, activated the p63 target genes (Figure S3C in Text S1). Altogether these data strongly suggest that this new p63 pathway is conserved in cervical squamous cell carcinoma and that the adhesion genes are modulated by TAp63β preferentially with no clear involvement of the ΔNp63 isoforms.

### HPV18 E6 modulates the TAp63β isoform by accelerating its degradation

**Figure 5:**
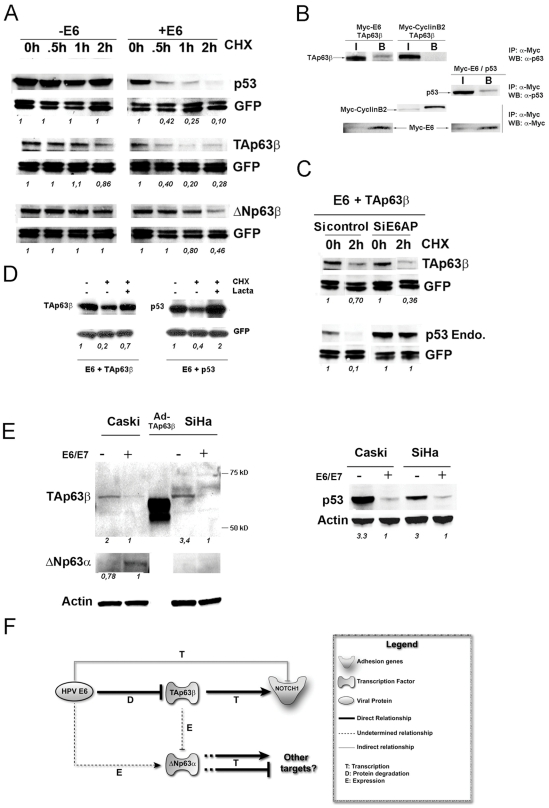
The HPV18 E6 oncogene interacts with and induces degradation of the TAp63β isoform in an E6AP independent manner. (A) TAp63β, ΔNp63β or p53 (as a positive control) were cotransfected with GFP expression vectors as controls for comparable transfection efficiency, in HeLa cells, in the presence or absence of a HPV18 E6 expression plasmid. After 24 h, cycloheximide (CHX) was added to the medium and cells were harvested at 0, 0.5, 1 or 2 h of treatment and analyzed by Western blotting with the p63, p53 and GFP antibodies as indicated. (B) Co-immunoprecipitation of cotransfected TAp63β and Myc-HPV18 E6 or Myc-Cyclin B2 as a negative control, were done in HeLa cells while cotransfection of Myc-HPV18 E6 and p53 was done as a positive control. (C) HeLa cells were transfected with control siRNA or E6AP siRNA for 20 h and were then cotransfected with TAp63β, GFP and HPV18 E6 expressing vectors. After 24 h, cells were treated with CHX for 2 h, harvested and analyzed by Western blot as described in panel A. E6AP siRNA efficiency was assessed through stabilization of the endogenous p53. (D) HeLa cells were co-transfected with HPV18 E6 and TAp63β or p53 as in panel A, with or without an additional treatment of 6 h by Lactacystin. (E) Caski and SiHa were either infected with E2 recombinant adenovirus to induce repression of E6/E7 transcription (-) or with the GFP (+). TA and ΔN p63 were detected by specific purified antibodies from Biolegend (Poly 6189) and the 4A4 from Pharmingen respectively, while p53 was detected as in panel B. (F) Model for the control of the p63 pathway in Caski cells. Quantification of the p53 and p63 expression, corrected by the GFP and actin controls, are given under each western blot picture with time 0 (panels A and C) or the controls (panels D and E) set up at 1.

We have shown that TAp63β is a transcriptional activator whose activity is repressed by the HPV oncogenes in Caski cells. Since expression of the viral oncogenes occurred through polycistronic mRNA as previously discussed, we could not separately repress E6 or E7 in our model system. However, the putative down modulation of TAp63β described here is reminiscent of the down modulation of p53 by E6 in cervical cancer [28]–[30], and we decided to verify the simpler hypothesis incriminating E6 rather than E7 in p63 modulation. We tested whether HPV18 E6 could degrade TAp63β in our system by cotransfection with an expression vector for HPV18 E6 into HeLa cells [29]. In the same experiment we also cotransfected p53 as a positive control and ΔNp63β to verify whether the TA isoform is preferentially degraded. We performed these experiments in the presence of cycloheximide to prevent protein synthesis. This revealed that expression of HPV18 E6 accelerated the degradation of TAp63β although often less efficiently than p53 (Figure 5A). The ΔNp63β isoform remained quite stable, at least for the first hour of cycloheximide treatment (Figure 5A). Thus E6 can induce degradation of the TAp63β isoform, whilst the ΔN isoform seems more resistant. Together with our finding that the TAp63β isoform is an activator of p63 target genes in Caski cells, its destabilization by E6, leading to transcriptional repression of the target genes, thus mimics the situation previously reported for p53 in cervical cancer.

The next step was to ask whether this degradation of TAp63β was mediated through interaction between TAp63β and E6 and via the ubiquitin ligase E6AP, as demonstrated for p53 [30]. Interaction between Myc-tagged E6 and TAp63β was readily detectable by coimmunoprecipitation in cotransfected HeLa cells treated with lactacystin to inhibit protein degradation. P53 was also coimmunoprecipitated with Myc-tagged E6, while the Myc-tagged Cyclin B, used as a negative control, did not coimmunoprecipitate with TAp63β (Figure 5B). We used an E6AP siRNA [31] to silence E6AP in HeLa cells cotransfected with E6 and TAp63β. Interestingly silencing of the E6AP ubiquitin ligase did not lead to stabilization of TAp63β (Figure 5C) while in the same cells, endogenous p53 was stabilized about 10-fold, back to its basal level in untreated cells (Figure 5C). This suggests the existence of another pathway for E6-mediated degradation of TAp63β which was however stabilized 3.5-fold by the proteasome inhibitor lactacystin as expected while p53 was stabilized 5-fold in the same experiment (Figure 5D).

We then needed to verify whether repression of E6 and E7 and subsequent stabilization of TAp63β could be detected in cervical carcinoma cell lines SiHa and Caski. We infected the two cell lines with the adenovirus expressing E2, as the best repressor of oncogenic transcription (repression of more than 90% compared to 60–70% of E6/E7 with siRNA) or adenovirus expressing GFP as a negative control. Extremely efficient repression of E6 and E7 induced a strong stabilization of 3-fold of the p53 protein, as expected, altogether with stabilization of 2 and 3.4-fold of the TAp63β isoform in Caski and SiHa respectively (Figure 5E). Ectopically expressed TAp63β appeared as a slightly lower migrating band due to insertion of a shorter version of the protein in the original expression vector as described [26] (Figure 5E). These data show that the low level of the TAp63β protein is due to its continuous degradation by E6, a situation reminiscent of p53 degradation. In the same Caski cells, we also observed slightly reduced expression (about 20% reduction) of the endogenous ΔNp63α isoform after E6/E7 repression and stabilization of TAp63β (Figure 5E) as demonstrated earlier in cells infected with the TAp63β recombinant adenovirus (Figure 4B, upper panel). However, this was not observed in SiHa cells where endogenous ΔNp63α is not detectable (Figure 5E). From these data we could draw a model whereby p63 target genes are activated by TAp63β which is targeted for degradation by E6; while ΔNp63α would modulate other target genes that are not studied here (Figure 5F). The overall effect of E6 expression in Caski is summarized by repression of the adhesion genes, represented by *notch1*, and activation of ΔNp63α, both by indirect mechanisms resulting from the pivotal degradation of TAp63β by E6 (Figure 5F).

### Repression of E6/E7 and expression of TAp63β increase focal adhesion in cervical carcinoma cells

**Figure 6:**
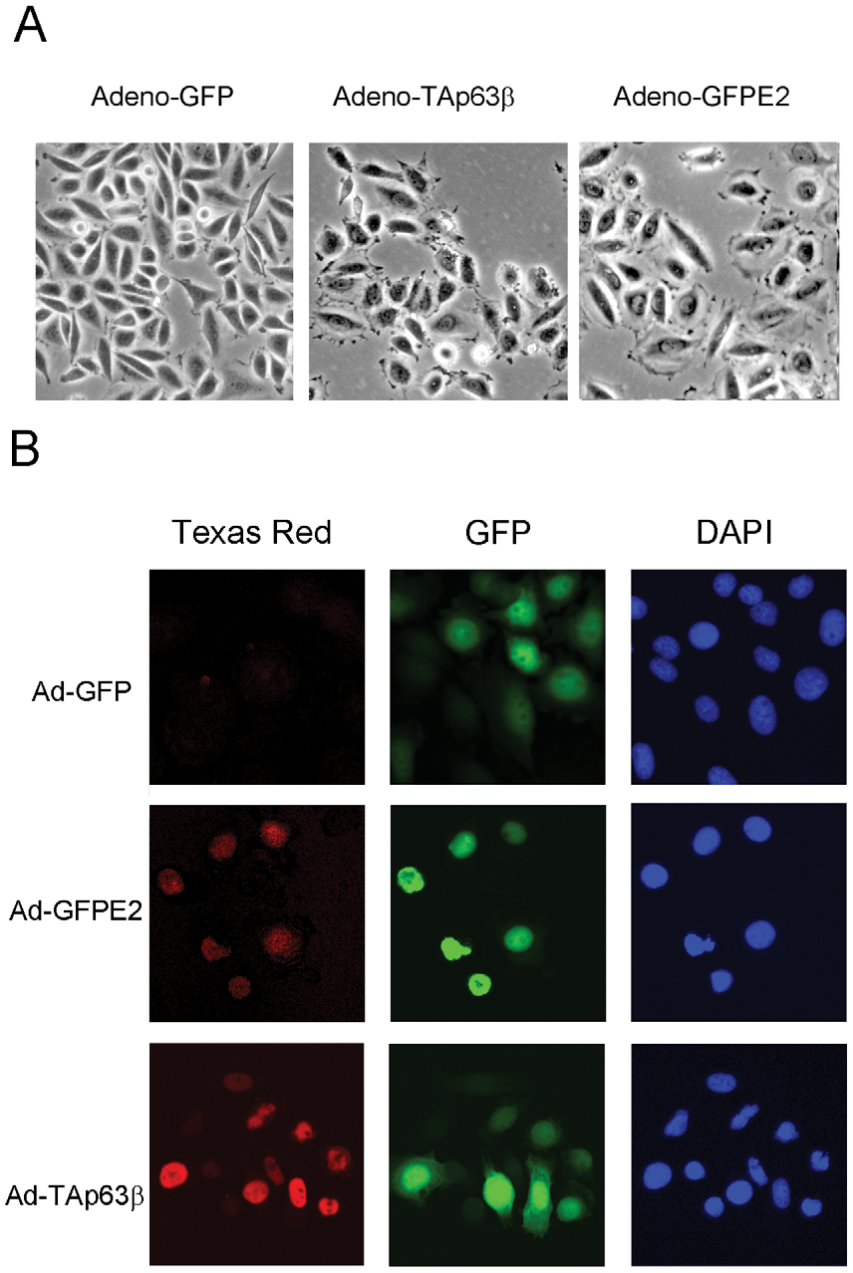
E6/E7 repression by E2 and ectopic expression TAp63β induce phenotypic changes in SiHa cells. (A) SiHa cells were either infected by recombinant adenoviruses expressing GFP alone, TAp63β or GFP-E2 as indicated at the top of the figure. Phase contrast microscopy was performed on live cells, 24 h after infection with TAp63β recombinant adenovirus and 42 h with the E2 recombinant adenovirus at 10X magnification. (B) Induction of TAp63 expression in SiHa cells infected by Ad-GFP-E2 that represses endogenous E6/E7 expression. Immunofluorescence of TAp63 is revealed by the purified specific antibody against this isoform (Biolegend, Poly 6189) counterstained with Texas red. The GFP expression controlling the infected cells is green while nuclei are stained in blue by DAPI.

**Figure 7:**
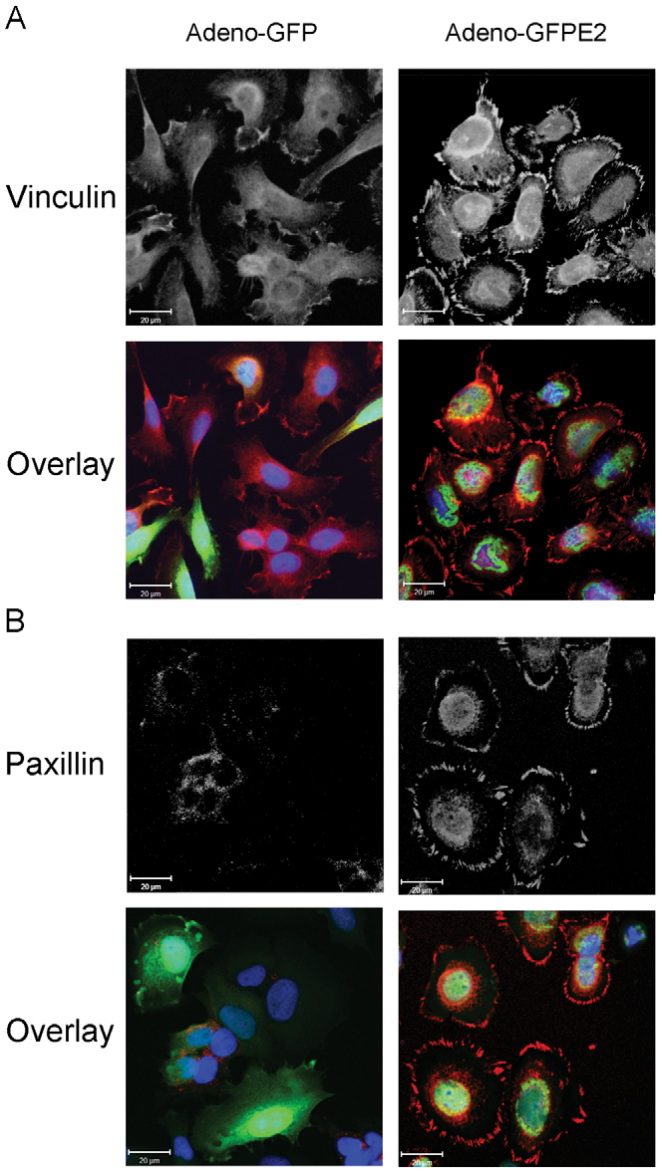
E6/E7 repression by E2 induces focal adhesion in SiHa cells. (A) SiHa cells grown on coverslips were infected by recombinant adenoviruses expressing either GFP (left panels) or GFP-E2 (right panels) at m.o.i. 100, in conditions where all the cells are infected and express green fluorescent protein. Staining with the monoclonal antibody against vinculin hVIN-1 (V9131, SIGMA) counterstained with anti-mouse Alexa is shown in upper panels. The overlay picture of vinculin in red, GFP in green and DNA in blue (DAPI) is shown in the lower panels. (B) Same as in (A) but with the monoclonal antibody against paxilin 5H11 (ab3127, Abcam). Pictures were taken with a Zeiss confocal laser scanning microscope at an original magnification of 40X.

**Figure 8:**
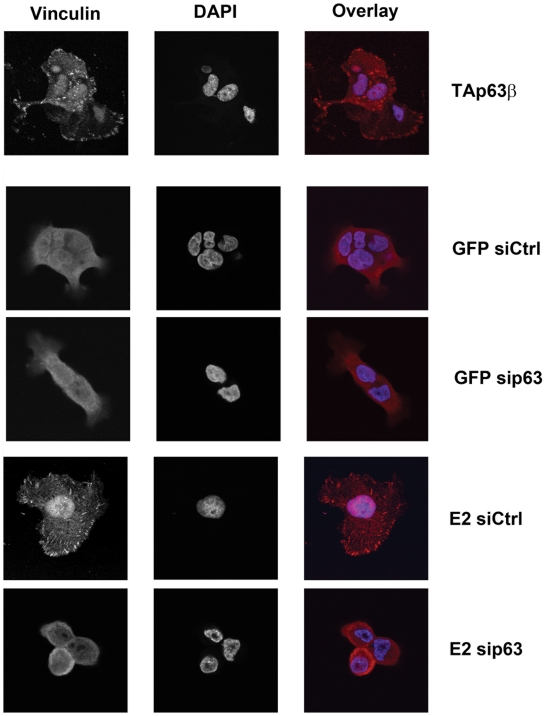
TAp63β ectopic expression and E6/E7 repression by E2 induce TAp63β-mediated focal adhesion in caski cells. Caski cells grown on cover slips were infected by recombinant adenoviruses expressing GFP, GFP-E2 or TAp63β at m.o.i. 100 as indicated, in conditions where all the cells are infected and express green fluorescent protein. Transfection with p63 siRNA was done in GFP and E2 infected cells as indicated. Staining with the monoclonal antibody against vinculin hVIN-1 (V9131, SIGMA) counterstained with anti-mouse Alexa is shown. The overlay pictures of vinculin in red and DNA in blue (DAPI) are shown. Pictures were taken with a Zeiss confocal laser scanning microscope at an original magnification of 40X.

The next question was whether activation of adhesion genes would stimulate phenotypic changes of TAp63β expressing cells. Cervical carcinoma SiHa cells infected with TAp63β or E2 recombinant adenoviruses acquired star-like structure together with enlargement of their cytoplasms (Figure 6A). In E2-expressing SiHa cells, these morphological changes correlated with stabilization of endogenous TAp63β as shown by immunofluorescence (Figure 6B) and western blot (Figure S5A in Text S1). Immunofluorescent labeling of vinculin and paxillin, markers of focal adhesion, showed an increase in number and size of the labeled foci at the membrane of E2-infected SiHa (Figure 7). In Caski cells, labeling of vinculin clearly showed an increase in focal adhesion in TAp63β and E2 expressing cells which was drastically reduced by silencing of p63 in E2 expressing cells, thus indicating that this increase in focal adhesion is mediated by TAp63 (Figure 8). An increase in focal adhesion was also seen in primary keratinocytes infected by the recombinant adenovirus expressing TAp63β, although these cells already present higher basal levels of focal adhesion than Caski (Figure S4 in Text S1), while in contrast no activation of TAp63β expression was detected in E2 expressing keratinocytes (Figure S5A in Text S1). Increase of focal adhesion and reorganization in cell membrane of E2 expressing SiHa and Caski cells were not accompanied by an increased expression of paxillin and involucrin, as shown by western blots (Figure S5B in Text S1). These results therefore point to an indirect role of TAp63β in modulating focal adhesion, which is in line with our previous findings since *paxillin* and *vincullin* were not found among the p63 transcriptional target genes. More work is needed however to fully characterize these phenotypic changes associated with expression of the TAp63β isoform and to understand the mechanism by which focal adhesion is modulated. Altogether, we have demonstrated here for the first time a strong involvement of the TAp63β isoform in formation of focal adhesion in epithelial cells and its down modulation by expression of the HPV E6 oncogene.

## Discussion

The p63 transcription factors are either activators or repressors of transcription depending on the isoforms expressed, the cell system and the target genes. However, repression of the viral oncogenes E6 and E7 in cervical carcinoma modulates p63 target genes in a unique way since an entire p63 target gene set was activated while none were repressed. It should be noted that activation of the p63 target genes in our system was generally lower than that of p53 target genes, as if the regulatory pathways leading to modulation of the p63 targets were less homogeneous. Indeed, p63 is expressed as various isoforms which can interact with each other and which share similar binding sites together with p53. This has led to an intricate situation where activators and repressors are not well defined. For instance, ΔNp63 is usually recognized as a repressor although it also contains a specific activator domain [32], [33], while TAp63 is mainly described as an activator due to the presence of the long N-terminal transactivation domain. However, the longest TAp63α isoform also contains a C-terminal domain which is a repressor of the TA domain, thus leading to inefficient transcriptional activation [34]. The most efficient activator so far described in the literature has been the shortest isoform TAp63γ but this was demonstrated with a group of p53 target genes [27] as confirmed in our system. In contrast, we demonstrated here that TAp63β can activate p63 target genes in a specific manner in Caski and SiHa cervical carcinoma cell lines. This finding is unexpected since the TAp63 isoforms have not been detected in human epithelial cells and had not been assigned specific roles in skin development [35], although TAp63 was recently shown to play an important role in regulating cellular senescence and genomic instability [21]. Its depletion in skin induces premature aging due to uncontrolled multiplication of skin stem cells which is in line with a putative oncogenic role of TAp63β depletion in cervical carcinomas.

Another major finding of our work is the fact that the HPV18 E6 oncogene can accelerate degradation of the TAp63β isoform, although independently of the E6AP ubiquitin ligase which is implicated in degradation of p53 [30]. TAp63 is known to be degraded through the proteasomal pathway via its amino-terminal TA domain [36], [37], independent of MDM2 [36]. The Itch ubiquitin ligase has been suggested as one of the major factors controlling the stability of both the TA and ΔN p63 isoforms [38] although it degrades the ΔN isoforms more efficiently [39], [40]. As for the putative roles of viral oncoproteins, it has been reported that they are unable to interact with p63 or to noticeably modify p63 activity [41]. Our work presents evidence that the high risk HPV18 E6 oncoprotein can bind TAp63β and accelerates its degradation. However and significantly, E6 uses a different pathway than for the degradation of p53 and the ubiquitin ligase involved in TAp63β degradation has yet to be identified. A striking consequence of this finding is that repression of the endogenous oncogenes induces stabilization of endogenous TAp63β together with p53, in all cervical carcinoma cell lines that we have examined including HeLa, Caski and SiHa. Another consequence of our findings is that ΔNp63 does not seem implicated in regulation of the anchorage independent growth in cervical cancer which is surprising in light of its pivotal role in skin homeostasis. In cervical carcinoma cells, stabilization of TAp63β induces an increase in focal adhesion. In keratinocytes, we could also show that overexpression of the β isoform increased focal adhesion. However the basal level of focal adhesion is higher in keratinocytes compared to cervical carcinoma cells and the best expressed TAp63 in these cells is the γ isoform. We infer from these data that the γ isoform is also probably able to modulate focal adhesion but more work is needed to clarify this point.

The p53/p63 family of transcription factors also includes p73 and its various isoforms [42], which share identical binding sites with p63 and can form heterotetrameric factors to concomitantly bind the same sites if expressed at the same time [43]. The p73 transcription factors however, are not expressed in skin and have no known roles in its homeostasis, while they play roles in neurogenesis, inflammation, sensory pathways and osteoblastic differentiation [44] as well as in genomic stability and tumor suppression [45]. Since the present study mostly describes modulation of a group of target genes involved in adhesion pathways that are therefore directly linked to skin maintenance and differentiation, it is presumed that p73 should not play a dramatic role here. We cannot exclude however that some cross-regulation could take place.

We have shown here that TAp63β activates genes involved in cell adhesion and induces phenotypic changes in focal adhesion in keratinocytes upon its ectopic expression. The bovine papillomavirus E6 protein has previously been shown to modulate focal adhesion through direct interaction with paxillin [46], [47]. We cannot exclude that HPV E6 could also directly influence cell adhesion in cervical carcinoma cells. However, we show here that E6 induces a strong modulation of focal adhesion through degradation of TAp63β. This novel role of E6 thereby participates in oncogenic transformation of cervical carcinoma cells through induction of anchorage independent growth. TAp63β therefore appears as a tumor suppressor in our system, which is in agreement with its recently reported roles in mouse models [21], [48], [49]. This is, to our knowledge, the first time that TAp63β is implicated in a human cancer, cervical carcinoma, which is one of the leading causes of death by cancer in women worldwide.

## Materials and Methods

### Infection of Caski and SiHa cells with recombinant adenoviruses

Recombinant adenoviruses expressing green fluorescent protein (GFP) alone, GFP fused to HPV18 E2 protein deleted of its transactivation domain (E2C) or E2 full-length were previously described [50]. Recombinant adenoviruses expressing TAp63β or ΔNp63β isoforms were prepared from the pIRES expression plasmids containing the TAp63β or ΔNp63β gene followed by the IRES sequence and the GFP gene. Recombinant adenoviruses expressing the TA and ΔNp63α isoforms were a kind gift of Barry Trink [26].

Cells were infected with purified adenoviruses expressing GFP, GFP-E2 or GFP-E2C at a multiplicity of infection (m.o.i.) of 100 or 200 PFU/cell or with adenoviruses expressing GFP, TAp63β or ΔNp63β at an m.o.i. of 20, 50 or 100 PFU/cell, as described previously [4].When needed, 24 hours after infection, cycloheximide was added at 100 µg/ml for up to 2 h and/or lactacystin was administered at 10 µM for up to 6 hours.

### Cell culture and transfections

HeLa, Caski and SiHa cells were grown using standard procedures in a 37°C humidified incubator with 5% CO2. All cell lines were grown in high-glucose Dulbecco's modified Eagle's medium (Invitrogen) with 10% heat-inactivated fetal bovine serum.

Plasmids were transfected using FuGENE HD (Roche) according to the manufacturer's instructions. Expression plasmids for the six p63 isoforms were kindly provided by Barry Trink.

For siRNA transfection, DharmaFECT 1 (Thermo Scientific) was used according to the manufacturer's instructions.

The siRNA sequences used were:

HPV18 E6/E7 siRNA: GCAUGGAGAUACACCUACAdTdT

HPV16 E6/E7 siRNA: GAGCUGCAAACAACUAUACdTdT

Control siRNAs used in HPV16 positive cells (Caski) and in HPV18 positive cells (HeLa) were HPV18 E6/E7 siRNA and HPV16 E6/E7 siRNA respectively.

For p63 siRNA (which targets all p63 isoforms) and p53 siRNA, we used ON-TARGETplus SMARTpools (Thermo Scientific), which are pools of 4 different siRNAs for each gene with the following sequences:

p63 siRNAs: (UCUAUCAGAUUGAGCAUUA; GCACACAGACAAAUGAUU; CGACAGUCUUGUACAAUUU; GAUGAACUGUUAUACUUAC)

p53 siRNAs: (GAAAUUUGCGUGUGGAGUA; GUGCAGCUGUGGGUUGAUU; GCAGUCAGAUCCUAGCGUC; GGAGAAUAUUUCACCCUUC).

### Microarray hybridization and analysis

The high density microarrays used in this study were the Human Gene 1.0 ST array from Affymetrix, including 28,869 well-annotated genes with 764,885 distinct probes. A total of 200 ng of RNA for each experiment was amplified, terminally labeled and used for hybridization according to the manufacturer's instructions (Affymetrix). The microarrays were washed and stained using Fluidics Station (GenChip) and scanned using a GeneArray 25000 Scanner. Data collected from each hybridization experiment were analyzed and statistical analyses carried out using Partek Genomics Suite software.

### RNA isolation and quantitative RT-PCR

Total RNA was extracted using RNAEasy Mini Kit (Qiagen) and a total of 2.5 µg of RNA were reverse transcribed with Superscript II (Invitrogen) according to the manufacturer's instructions. From the resulting synthesized single-stranded cDNA, 1/100 was used for each Real-time PCR in the presence of 1 µM of specific primers and Syber Green master mix (Applied Biosystems). Quantitative PCR were performed with an MX3005P sequence detection system (Stratagene). Each cDNA was normalized to histone deacetylase 1 (HDAC1), glyceraldehyde-3-phosphate dehydrogenase (GADPH), and 18S ribosomal RNA. PCR were performed in duplicate and fitted to standard curves, providing mean cycle threshold values that were translated into arbitrary units corresponding to mRNA levels. Data were analyzed with MxPro v.4.00 software (Stratagene).

Primers:

GADPH: 5′-TCCATCACCATCTTCCAGG-3′; 5′-CATCGCCCCACTTGATTTTG-3′

HDAC: 5′-TTTTCAAGCCGGTCATGTCC-3′; 5′- CCGCACTAGGCTGGAACATC-3′

18S: 5′-TGCGAATGGCTCATTAAATCAGT-3′; 5′-AGAGGAGCGAGCGACCAA-3′

16E6E7: 5′-GCTCAGAGGAGGAGGATGAAATAG-3′; 5′-TCCGGTTCTGCTTGTCCAG-3′

18E6E7: 5′-CCCCAAAATGAAATTCCGGT-3′; 5′-GTCGCTTAATTGCTCGTGACATA-3′

pan-p63: 5′-GACAGGAAGGCGGATGAAGATAG-3′; 5′-TGTTTCTGAAGTAAGTGCTGGTGC-3′

TAp63: 5′-AAGATGGTGCGACAAACAAG-3′; 5′-AGAGAGCATCGAAGGTGGAG -3′

ΔNp63: 5′-TCAATTTAGTGAGCCACAGTAC-3′; 5′-CTGTGTTATAGGGACTGGTG-3′

p53: 5′-TTCGACATAGTGTGGTGGTGC-3′; 5′-AGTCAGAGCCAACCTCAGGC-3′

CENPA: 5′-TCAGAGTAGCCTCACCATTAGTGG-3′; 5′-AGCTACACATCCGTTGACAAGC-3′

CTGF: 5′-CCCTGCATCTTCGGTGGTA-3′; 5′-AGGCACGTGCACTGGTACTTG-3′

ITGA2: 5′- CTTTGGTTAGCCTTGCCTTAGG-3′; 5′- CCCCTGCAAGGAAGAATCAC-3′

LAMB3: 5′-GGGCACTCAGAGACATGTCACTT-3′; 5′- TGACACCGCTCACAGTTCTTG-3′

CEACAM1: 5′-CCAAATCAAAGCCAGCAAGACCAC-3′; 5′-TCATTTGTGGAGCAGGTCAGGTTC-3′

Notch1: 5′- GAGTGGGACCAACTGTGACAT-3′; 5′-CCGTTGACACAAGGGTT-3′

FAT: 5′- CAACCTTCCCTACTACGCCG-3′; 5′- ACATGGCCCACCTCAGTGTC-3′

FN1: 5′-GTGTGTTGGGAATGGTCGTG-3′; 5′-AAGCTGCGAGTAGGCAATGC-3′

p21: 5′-GCGACTGTGATGCGCTAATG-3′; 5′-CGGTGACAAAGTCGAAGTTCC-3′

### Statistics

All Real time PCR data are represented as mean ± SD. Data were analyzed using paired t test for comparison between two groups. P Values under 0.05 were considered significant. All experiments were done at least three times.

### Western blot

Infected or transfected cells were collected 24 or 42 h post-infection or post-transfection, and protein extracts were subjected to electrophoresis before transfer onto nitrocellulose membranes. The membranes were then incubated with antibodies specific for: p63 (4A4, BD Pharmingen), TAp63 purified antibody (Biolegend Poly 6189) or the monoclonal TAp63, a kind gift of Franck McKeon, [35], p53 (DO-1, Santa-Cruz), GFP (TP401; Torrey Pines Biolabs) or beta-actin (A2066, Sigma); and either mouse or rabbit secondary antibodies coupled to peroxidase. Protein bands were revealed using the Amersham ECL plus kit. Quantification of western blots was done by measuring the relative intensity of the bands compared to internal controls (GFP and or actin), the values given are in arbitrary units.

### Co-immunoprecipitation

Hela cells were co-transfected with TAp63β and Myc-E6 HPV18 or Myc-CyclinB2, as a negative control. As positive control, HeLa cells were cotransfected with Myc-E6 HPV18 and p53 expressing vectors. 20 h post-transfection, cells were treated with 10 µM of lactacystin for 6 h. Cells were harvested and proteins were extracted in an extraction buffer (300 mM NaCl, 50 mM Tris-HCl [pH 8], 0.5 % Nonidet P-40 [NP-40], 1 mM EDTA, protease and phosphatase inhibitors) for 30 min at 4°C, followed by centrifugation. Extracts containing 1 mg of total proteins were immunoprecipitated with the rabbit anti-myc antibody (A-14; Santa Cruz) 5 h at 4°C. After centrifugation, beads were washed four times with 1 ml of extraction buffer. Proteins from the beads and input (1/40 of total proteins) were separated by sodium dodecyl sulfate-polyacrylamide gel electrophoresis (SDS-PAGE) and analyzed in Western blot experiments. For Western blot experiments, mouse anti-Myc (9E10; Santa Cruz), mouse anti-p53 (DO-1; Santa Cruz) and mouse anti-pan-p63 (4A4, BD Pharmingen) antibodies were used.

### Immunofluorescence

Cells grown on cover slips were fixed at 4°C for 30 min with 2% paraformaldehyde (v/v) and washed in PBS. Cells were then permeabilized for 30 min at room temperature in PBS, 2% serum, 0.1% triton. After washing with PBS, the cells were incubated with primary antibodies for 1 h at room temperature, washed 3 times with PBS, 2% serum, followed by secondary antibodies for 1 h at room temperature. After washing with PBS, cell nuclei were stained with DAPI (Invitrogen). Antibodies against paxillin (clone 5H11, Abcam ab3127) and vinculin (clone hVIN-1, Sigma) and TAp63 purified antibody (Biolegend Poly 6189) were used. Secondary antibodies were fused to Alexa fluor 568 (Invitrogen). Confocal fluorescent images were obtained by a Zeiss confocal laser scanning microscope with a 40X objective.

### Accession numbers

p63: *603273, p53: *191170, pRb: +180200, p73: *601990, E2F1: *189971, p57kip2: *600856, p21: *116899, CENPA: *117139, CTGF: *121009, ITGA2: +192974, CEACAM1: *109770, Notch1: *190198, COL5A1: *120215, LAMB2: *150325, FAT: *600976, FN1: +135600, FBN1: *134797, GDF15: *605312, Sestrin: *606103, RRM2B: *604712, E6AP: *601623, Paxillin: *602505, Vinculin: *193065, MDM2: +164785, GADPH: *138400, HDAC: *601241, 18S: *180473.

## Supporting Information

Table S1 Common genes found activated by repression of E6/E7 in Caski cells in the microarrays and in the p53 ChIP-chip data [1]–[3] using TRANSFAC Professional software (Biobase). Values given are ratio of mRNA levels in E2C expressing cells compared to GFP expressing cells.
(PDF)

Table S2 Common genes found activated by repression of E6/E7 in Caski cells in the microarrays and in the p63 ChIP-chip data [4] using TRANSFAC Professional software (Biobase). Values are given as in Table S1.
(PDF)

Text S1 Supplementary material contains five figures (Figures S1 to S5) and one Table S3 and their respective legends and references for all supplementary material.
(DOC)
