# Supplementary material for: The Human Papillomavirus E6 Oncogene Represses a Cell Adhesion Pathway and Disrupts Focal Adhesion through Degradation of TAp63β upon Transformation
Source: PLoS Pathog. 2011 Sep 29;7(9):e1002256. doi: 10.1371/journal.ppat.1002256 (PMC3182928; doi:10.1371/journal.ppat.1002256)
Supplement: Table S1 — Common genes found activated by repression of E6/E7 in Caski cells in the microarrays and in the p53 ChIP-chip data [1]–[3] using TRANSFAC Professional software (Biobase). Values given are ratio of mRNA levels in E2C expressing cells compared to GFP expressing cells. (PDF) [file ppat.1002256.s001.pdf]

© 2000-2009 Ingenuity Systems, Inc. All rights reserved.

p-value	Fold Change	Symbol	Entrez Gene Name
1.07E-03	1.691	AEN	apoptosis enhancing nuclease
6.63E-03	1.562	ANK1	ankyrin 1, erythrocytic
4.04E-04	1.779	ARSA	arylsulfatase A
7.12E-05	1.760	BAX	BCL2-associated X protein
6.87E-03	1.655	BNIP3 (includes E	BCL2/adenovirus E1B 19kDa interacting protein 3
1.52E-03	1.534	C6ORF155	chromosome 6 open reading frame 155
2.27E-03	2.097	C6ORF204	chromosome 6 open reading frame 204
1.86E-03	1.837	CALD1	caldesmon 1
4.87E-04	1.694	CCDC51	coiled-coil domain containing 51
4.76E-05	3.694	CDKN1A	cyclin-dependent kinase inhibitor 1A (p21, Cip1)
3.15E-05	1.834	CES2 (includes E	carboxylesterase 2 (intestine, liver)
1.29E-05	3.282	CROT	carnitine O-octanoyltransferase
5.43E-05	1.525	DDB2	damage-specific DNA binding protein 2, 48kDa
9.79E-04	1.506	DDIT4	DNA-damage-inducible transcript 4
6.33E-05	1.745	DNAH11	dynein, axonemal, heavy chain 11
1.32E-03	1.507	FAM3C	family with sequence similarity 3, member C
8.78E-05	1.664	FAT1	FAT tumor suppressor homolog 1 (Drosophila)
4.59E-03	1.587	FGF1	fibroblast growth factor 1 (acidic)
1.17E-03	1.557	FST	follistatin
3.35E-05	1.756	GLS2	glutaminase 2 (liver, mitochondrial)
2.37E-03	1.503	GNAI1	guanine nucleotide binding protein (G protein), alpha inhibiting activity polypeptide 1
1.17E-04	2.038	KIAA1370	KIAA1370
1.03E-04	2.255	KITLG	KIT ligand
1.44E-06	1.849	LIMK2	LIM domain kinase 2
1.50E-03	1.784	LYST	lysosomal trafficking regulator
5.66E-04	2.084	NAV3	neuron navigator 3
1.98E-04	1.841	NDRG1	N-myc downstream regulated 1
7.28E-04	1.809	NOTCH1	Notch homolog 1, translocation-associated (Drosophila)
6.88E-05	1.556	PGPEP1	pyroglutamyl-peptidase I
9.17E-03	2.205	PLA2G4A	phospholipase A2, group IVA (cytosolic, calcium-dependent)
3.84E-04	2.056	PRDM1	PR domain containing 1, with ZNF domain
1.49E-03	2.389	PSTPIP2	proline-serine-threonine phosphatase interacting protein 2
3.08E-04	3.033	PTGS2	prostaglandin-endoperoxide synthase 2 (prostaglandin G/H synthase and cyclooxygenase)
1.65E-03	1.795	PTPRE	protein tyrosine phosphatase, receptor type, E
1.17E-03	1.804	PTPRM	protein tyrosine phosphatase, receptor type, M

1.25E-05	3.190	RPS27L (includes	ribosomal protein S27-like
4.79E-06	3.078	RRM2B	ribonucleotide reductase M2 B (TP53 inducible)
4.13E-05	3.603	SESN1	sestrin 1
3.05E-04	1.995	SLC12A4	solute carrier family 12 (potassium/chloride transporters), member 4
3.90E-04	1.508	STX6	syntaxin 6
3.81E-04	1.779	SULF2	sulfatase 2
7.59E-04	1.939	TGFA	transforming growth factor, alpha
5.60E-05	2.252	TLR3	toll-like receptor 3
1.94E-04	1.546	TMEM50B	transmembrane protein 50B
3.38E-05	2.273	TNFRSF10B	tumor necrosis factor receptor superfamily, member 10b
6.61E-07	3.117	TNFSF9	tumor necrosis factor (ligand) superfamily, member 9
1.26E-06	6.253	TRIM22	tripartite motif-containing 22
3.90E-03	1.755	TRIM5	tripartite motif-containing 5
1.38E-03	1.639	TTYH3	tweety homolog 3 (Drosophila)
7.96E-05	2.652	XPC	xeroderma pigmentosum, complementation group C
7.71E-04	1.584	XPR1	xenotropic and polytropic retrovirus receptor
1.81E-04	3.310	ZMAT3	zinc finger, matrin type 3
