# Supplementary material for: The Human Papillomavirus E6 Oncogene Represses a Cell Adhesion Pathway and Disrupts Focal Adhesion through Degradation of TAp63β upon Transformation
Source: PLoS Pathog. 2011 Sep 29;7(9):e1002256. doi: 10.1371/journal.ppat.1002256 (PMC3182928; doi:10.1371/journal.ppat.1002256)
Supplement: Table S2 — Common genes found activated by repression of E6/E7 in Caski cells in the microarrays and in the p63 ChIP-chip data [4] using TRANSFAC Professional software (Biobase). Values are given as in Table S1. (PDF) [file ppat.1002256.s002.pdf]

© 2000-2009 Ingenuity Systems, Inc. All rights reserved.

p-value	Fold Change	Symbol	Entrez Gene Name
9.28E-06	3.427	ABCA12	ATP-binding cassette, sub-family A (ABC1), member 12
7.07E-04	2.676	ACER2	alkaline ceramidase 2
2.99E-03	1.748	ACTG2 (includes	actin, gamma 2, smooth muscle, enteric
4.16E-05	1.833	ADAM19	ADAM metallopeptidase domain 19 (meltrin beta)
1.07E-03	1.691	AEN	apoptosis enhancing nuclease
2.60E-05	2.242	ANKRA2	ankyrin repeat, family A (RFXANK-like), 2
2.14E-03	1.664	ANKRD42	ankyrin repeat domain 42
4.78E-04	2.543	ANTXR2	anthrax toxin receptor 2
1.23E-04	1.730	ANXA4	annexin A4
3.39E-04	1.853	ARAP1	ArfGAP with RhoGAP domain, ankyrin repeat and PH domain 1
1.61E-04	4.782	AREG	amphiregulin
1.79E-04	4.286	AREG	amphiregulin
4.67E-04	1.798	ATG4A	ATG4 autophagy related 4 homolog A (S. cerevisiae)
4.04E-05	1.585	BCL2L1	BCL2-like 1
7.36E-03	2.032	BLOC1S2	biogenesis of lysosomal organelles complex-1, subunit 2
3.54E-05	1.621	BLZF1	basic leucine zipper nuclear factor 1
6.87E-03	1.655	BNIP3 (includes E	BCL2/adenovirus E1B 19kDa interacting protein 3
9.47E-03	1.794	BTBD10	BTB (POZ) domain containing 10
3.76E-03	1.530	BTBD11	BTB (POZ) domain containing 11
4.24E-05	3.319	BTG2	BTG family, member 2
2.27E-03	2.097	C6ORF204	chromosome 6 open reading frame 204
9.84E-03	1.604	C7ORF60	chromosome 7 open reading frame 60
2.04E-04	1.891	CALCOCO1	calcium binding and coiled-coil domain 1
1.86E-03	1.837	CALD1	caldesmon 1
9.03E-03	1.675	CASP1	caspase 1, apoptosis-related cysteine peptidase (interleukin 1, beta, convertase)
1.66E-03	1.594	CBLB	Cas-Br-M (murine) ecotropic retroviral transforming sequence b
4.87E-04	1.694	CCDC51	coiled-coil domain containing 51
4.22E-03	1.547	CD59	CD59 molecule, complement regulatory protein
4.76E-05	3.694	CDKN1A	cyclin-dependent kinase inhibitor 1A (p21, Cip1)
3.15E-05	1.834	CES2 (includes E	carboxylesterase 2 (intestine, liver)
4.39E-04	1.552	CFH	complement factor H
5.71E-04	1.885	CLCA2 (includes	chloride channel accessory 2
2.15E-03	1.835	COBLL1	COBL-like 1
1.03E-04	1.930	COL4A5	collagen, type IV, alpha 5
5.62E-04	2.567	CPEB4	cytoplasmic polyadenylation element binding protein 4

1.29E-05	3.282	CROT	carnitine O-octanoyltransferase
1.16E-03	1.767	CSF1	colony stimulating factor 1 (macrophage)
7.31E-04	1.991	CSNK1G1	casein kinase 1, gamma 1
8.53E-03	2.105	CTGF	connective tissue growth factor
3.75E-06	1.817	CUL9	cullin 9
5.45E-03	1.542	CYR61	cysteine-rich, angiogenic inducer, 61
5.43E-05	1.525	DDB2	damage-specific DNA binding protein 2, 48kDa
9.79E-04	1.506	DDIT4	DNA-damage-inducible transcript 4
2.87E-03	1.753	DENND2C	DENN/MADD domain containing 2C
4.78E-05	1.866	DGKA	diacylglycerol kinase, alpha 80kDa
6.33E-05	1.745	DNAH11	dynein, axonemal, heavy chain 11
1.37E-05	1.849	DOCK4	dedicator of cytokinesis 4
8.42E-04	2.764	DQX1	DEAQ box RNA-dependent ATPase 1
2.66E-04	1.864	DRAM (includes	damage-regulated autophagy modulator
4.53E-04	2.235	DUSP1	dual specificity phosphatase 1
4.82E-04	1.772	DUSP14	dual specificity phosphatase 14
5.46E-04	2.005	DUSP6	dual specificity phosphatase 6
9.25E-04	1.728	EMP1	epithelial membrane protein 1
3.26E-04	1.926	EPAS1	endothelial PAS domain protein 1
7.54E-04	1.681	ERRFI1	ERBB receptor feedback inhibitor 1
1.07E-03	2.273	ETV5	ets variant 5
1.11E-03	1.839	FAM174A	family with sequence similarity 174, member A
2.04E-03	1.688	FAM69A	family with sequence similarity 69, member A
8.78E-05	1.664	FAT1	FAT tumor suppressor homolog 1 (Drosophila)
5.83E-03	1.502	FAT4	FAT tumor suppressor homolog 4 (Drosophila)
1.20E-04	2.181	FBXW7	F-box and WD repeat domain containing 7
3.83E-04	2.139	FDXR	ferredoxin reductase
4.59E-03	1.587	FGF1	fibroblast growth factor 1 (acidic)
1.17E-03	1.557	FST	follistatin
1.05E-03	2.470	FYB	FYN binding protein (FYB-120/130)
1.02E-04	2.293	GALNT5	UDP-N-acetyl-alpha-D-galactosamine:polypeptide N-acetylgalactosaminyltransferase 5 (GalNAc-T5)
5.70E-04	1.791	GM2A	GM2 ganglioside activator
2.37E-03	1.503	GNAI1	guanine nucleotide binding protein (G protein), alpha inhibiting activity polypeptide 1
5.88E-04	1.636	GPR87	G protein-coupled receptor 87
3.29E-03	1.917	HAS3	hyaluronan synthase 3
1.54E-04	1.822	HBEGF	heparin-binding EGF-like growth factor
4.94E-04	1.933	HHAT	hedgehog acyltransferase

1.32E-03	1.569	HIST1H2BK	histone cluster 1, H2bk
3.84E-06	1.857	HSPG2 (includes	heparan sulfate proteoglycan 2
7.93E-03	1.547	IGFBP7	insulin-like growth factor binding protein 7
1.42E-03	1.539	IP6K2	inositol hexakisphosphate kinase 2
1.95E-04	1.997	ISCU	iron-sulfur cluster scaffold homolog (E. coli)
1.12E-04	1.712	ITGA2	integrin, alpha 2 (CD49B, alpha 2 subunit of VLA-2 receptor)
1.38E-04	1.647	ITPR2	inositol 1,4,5-triphosphate receptor, type 2
5.11E-03	1.547	IVL	involucrin
1.18E-04	2.106	KIAA0040	KIAA0040
1.30E-03	1.504	KIAA0247	KIAA0247
2.20E-04	1.605	KIAA1432	KIAA1432
1.03E-04	2.255	KITLG	KIT ligand
8.74E-04	1.620	LAMB3	laminin, beta 3
6.20E-03	1.564	LCE1B (includes	late cornified envelope 1B
4.62E-04	1.640	LDB1	LIM domain binding 1
1.50E-03	1.784	LYST	lysosomal trafficking regulator
9.57E-03	1.511	MACF1	microtubule-actin crosslinking factor 1
4.20E-03	1.716	MAGED2	melanoma antigen family D, 2
4.17E-05	2.255	MAP3K13	mitogen-activated protein kinase kinase kinase 13
7.10E-04	1.623	MAPKBP1	mitogen-activated protein kinase binding protein 1
4.06E-04	1.561	MAST4	microtubule associated serine/threonine kinase family member 4
6.53E-05	4.702	MDM2	Mdm2 p53 binding protein homolog (mouse)
6.93E-06	1.994	MMP14	matrix metallopeptidase 14 (membrane-inserted)
6.36E-04	1.619	MMP28	matrix metallopeptidase 28
1.60E-05	2.631	MR1	major histocompatibility complex, class I-related
3.16E-04	1.539	MYO1E	myosin IE
1.98E-04	1.841	NDRG1	N-myc downstream regulated 1
9.21E-04	2.602	NID1	nidogen 1
4.19E-03	1.918	NT5E	5'-nucleotidase, ecto (CD73)
6.35E-04	1.518	ORAI3	ORAI calcium release-activated calcium modulator 3
1.15E-05	1.661	P4HA2	prolyl 4-hydroxylase, alpha polypeptide II
2.17E-03	1.606	PCMTD1	protein-L-isoaspartate (D-aspartate) O-methyltransferase domain containing 1
6.88E-05	1.556	PGPEP1	pyroglutamyl-peptidase I
1.11E-04	1.602	PION	pigeon homolog (Drosophila)
9.17E-03	2.205	PLA2G4A	phospholipase A2, group IVA (cytosolic, calcium-dependent)
1.35E-04	2.355	PLAC8	placenta-specific 8
1.37E-04	1.943	PLAT	plasminogen activator, tissue

3.61E-06	2.045	POLH	polymerase (DNA directed), eta
1.69E-05	1.522	PPFIBP1	PTPRF interacting protein, binding protein 1 (liprin beta 1)
3.84E-04	2.056	PRDM1	PR domain containing 1, with ZNF domain
1.28E-04	1.738	PRKX	protein kinase, X-linked
3.48E-04	1.657	PROM2	prominin 2
3.08E-04	3.033	PTGS2	prostaglandin-endoperoxide synthase 2 (prostaglandin G/H synthase and cyclooxygenase)
1.65E-03	1.795	PTPRE	protein tyrosine phosphatase, receptor type, E
2.75E-04	1.850	PTPRZ1	protein tyrosine phosphatase, receptor-type, Z polypeptide 1
9.85E-03	1.525	RAB7A	RAB7A, member RAS oncogene family
3.37E-03	1.501	RASAL2	RAS protein activator like 2
6.30E-05	1.992	RDH10	retinol dehydrogenase 10 (all-trans)
4.41E-06	1.841	RETSAT	retinol saturase (all-trans-retinol 13,14-reductase)
1.35E-04	1.707	RGS12	regulator of G-protein signaling 12
1.26E-03	1.614	RHOC	ras homolog gene family, member C
3.50E-03	1.727	RIC3 (includes E	resistance to inhibitors of cholinesterase 3 homolog (C. elegans)
1.25E-05	3.190	RPS27L (includes	ribosomal protein S27-like
4.79E-06	3.078	RRM2B	ribonucleotide reductase M2 B (TP53 inducible)
2.94E-04	2.395	SCGB1A1	secretoglobin, family 1A, member 1 (uteroglobin)
3.17E-04	1.574	SEC31A	SEC31 homolog A (S. cerevisiae)
3.77E-03	1.503	SERPINB5	serpin peptidase inhibitor, clade B (ovalbumin), member 5
4.13E-05	3.603	SESN1	sestrin 1
1.24E-03	1.738	SIPA1L2	signal-induced proliferation-associated 1 like 2
1.92E-03	2.095	SLAMF7	SLAM family member 7
2.03E-04	1.747	SLC16A12	solute carrier family 16, member 12 (monocarboxylic acid transporter 12)
1.92E-04	1.690	SLC17A5	solute carrier family 17 (anion/sugar transporter), member 5
5.49E-05	1.510	SLC20A2	solute carrier family 20 (phosphate transporter), member 2
3.07E-04	1.602	SLC9A1	solute carrier family 9 (sodium/hydrogen exchanger), member 1
7.58E-05	2.580	SPATA18	spermatogenesis associated 18 homolog (rat)
8.32E-04	1.601	STK17A	serine/threonine kinase 17a
3.90E-04	1.508	STX6	syntaxin 6
3.81E-04	1.779	SULF2	sulfatase 2
2.67E-03	1.676	TCTEX1D2	Tctex1 domain containing 2
7.59E-04	1.939	TGFA	transforming growth factor, alpha
5.60E-05	2.252	TLR3	toll-like receptor 3
1.57E-03	1.659	TMEM131	transmembrane protein 131
2.15E-03	1.501	TMEM27	transmembrane protein 27
1.94E-04	1.546	TMEM50B	transmembrane protein 50B

9.85E-05	2.256	TMEM63B	transmembrane protein 63B
4.17E-04	1.602	TMEM68	transmembrane protein 68
2.38E-04	1.720	TNFAIP3	tumor necrosis factor, alpha-induced protein 3
3.38E-05	2.273	TNFRSF10B	tumor necrosis factor receptor superfamily, member 10b
6.61E-07	3.117	TNFSF9	tumor necrosis factor (ligand) superfamily, member 9
2.15E-04	2.025	TOB1	transducer of ERBB2, 1
2.73E-06	7.914	TP53INP1	tumor protein p53 inducible nuclear protein 1
5.02E-04	3.348	WDR63	WD repeat domain 63
7.96E-05	2.652	XPC	xeroderma pigmentosum, complementation group C
7.71E-04	1.584	XPR1	xenotropic and polytropic retrovirus receptor
1.17E-03	1.958	ZFYVE1	zinc finger, FYVE domain containing 1
1.81E-04	3.310	ZMAT3	zinc finger, matrin type 3
8.02E-04	1.744	ZNF385A	zinc finger protein 385A
