# Supplementary material for: The Human Papillomavirus E6 Oncogene Represses a Cell Adhesion Pathway and Disrupts Focal Adhesion through Degradation of TAp63β upon Transformation
Source: PLoS Pathog. 2011 Sep 29;7(9):e1002256. doi: 10.1371/journal.ppat.1002256 (PMC3182928; doi:10.1371/journal.ppat.1002256)
Supplement: Text S1 — Supplementary material contains five figures (Figures S1 to S5) and one Table S3 and their respective legends and references for all supplementary material. (DOC) [file ppat.1002256.s003.doc]

**Text S1**

Supplementary material contains five figures (Figures S1 to S5) and one table S3 and their respective legends and references for all supplementary material.

FIGURE S1.

E6/E7 repression induces a strong response of cellular adhesion genes in Caski cells.

Real-time PCR analyses using Human Extracellular Matrix and Adhesion Molecules PCR Array (SABiosciences), were carried out on 84 adhesion genes (including genes encoding collagens, laminins, metallopeptidases and integrins) in Caski cell infected with adenoviruses expressing GFP-E2C compared to GFP (A) or transfected with E6/E7 siRNA compared to control siRNA (B). Values given are ratios of mRNA levels in GFP-E2C compared to GFP infected cells (A) or in E6/E7 siRNA compared to control siRNA transfected cells (B). The list of genes can be found at the website of the manufacturer ([www.sabiosciences.com/rt_pcr_product/HTML/PAHS-013A.html](http://www.sabiosciences.com/rt_pcr_product/HTML/PAHS-013A.html)). Each individual gene can be found by using the numbers and letters corresponding to the 96 wells plate.

Shade of blue color used for the bars is to help reading the 3-dimentional data.

FIGURE S2

TAp63 α and β isoforms activate the transcription of a set of p63 target genes, while TAp63 γ activates transcription of p53 target genes in HeLa cells.

HeLa cells were transfected with expression vectors of the six p63 isoforms [5] or of p53 and Real-time PCR analyses were performed on the previously described group of endogenous p63 target genes [6] with cells transfected by GFP compared to TAp63 isoforms (A), ΔNp63 isoforms (B) or p53 (C). Modulation of p53 target genes in HeLa cells transfected with the six p63 isoforms compared to GFP, was assayed by Real-time PCR (D). Values given are ratios of mRNA levels in p63 or p53 transfected cells compared to GFP transfected cells (mean ± SD) and the threshold at 1.5 is indicated by the horizontal bars.

FIGURE S3

A set of adhesion genes is modulated in the HPV16-associated SiHa cells.

(A) SiHa were infected with the recombinant adenovirus expressing E2C compared to GFP and the mRNA levels of endogenous E6/E7 and p63 target genes were studied by real-time PCR. Levels of mRNA were given in arbitrary units (a.u.) with the endogenous levels in GFP infected cells set up at 1000 (mean ± SD, *p<0.05). (B) SiHa cells were infected with recombinant adenoviruses expressing TAp63β and ΔNp63β compared to GFP. Levels of p63 mRNA expression in SiHa cells are given as Ct values of real time PCR using the pan p63 primers, which can amplify all the p63 isoforms (mean ± SD, *p<0.05, higher Ct values correspond to lower mRNA levels). (C) TAp63β activates the endogenous p63 target genes in SiHa cells but not ΔNp63β after adenoviral infection with p63 recombinant adenoviruses. mRNA levels were obtained by RT-PCR and calculated as in panel A (mean ± SD, *p<0.05).

FIGURE S4

Ectopic expression of TAp63β increases focal adhesion in primary keratinocytes.

(A) Primary human keratinocytes grown on coverslips were infected by recombinant adenoviruses expressing either GFP (left panels) or TAp63β (right panels) at m.o.i. 50, in conditions where all the cells are infected and express green fluorescent protein. Staining with the monoclonal antibody against vinculin hVIN-1 (V9131, SIGMA) counterstained with anti-mouse Alexa is shown in upper panels. The overlay picture of vinculin in red, GFP in green and DNA in blue (DAPI) is shown in the lower panels. (B) Same as in (A) but with the monoclonal antibody against paxilin 5H11 (ab3127, Abcam). Original magnification was 63X.

FIGURE S5

Expression of p63 in human primary keratinocytes and SiHa and expression of paxillin and vincullin in Caski and SiHa cells infected by recombinant adenoviruses expressing GFP alone or GFP-E2. (A) Western blot analyses of induction of TAp63β in SiHa (HPV16-associated) cervical carcinoma cell line after E2-induced repression of E6/E7 oncogenes. TAp63β is expressed at very low levels in human primary keratinocytes (HPK) expressing either GFP or E2. High expression of ΔNp63α is detected only in HPK cells. (B) Vinculin and paxillin are constitutively expressed in Caski and SiHa, independent of the phenotypic changes induced by expression of E2.

Table S1

Common genes found activated by repression of E6/E7 in Caski cells in the microarrays and in the p53 ChIP-chip data [1-3] using TRANSFAC Professional software (Biobase). Values given are ratio of mRNA levels in E2C expressing cells compared to GFP expressing cells.

Table S2

Common genes found activated by repression of E6/E7 in Caski cells in the microarrays and in the p63 ChIP-chip data [4] using TRANSFAC Professional software (Biobase). Values are given as in Table S1.

Table S3

Genes activated by repression of E6/E7 in Caski cells in the microarrays and belonging to adhesion pathway. Values given are ratio of mRNA levels in E2C expressing cells compared to GFP expressing cells. Asterisks indicate genes that were further studied.

REFERENCES for supplementary data

1. Kapranov P, Cawley SE, Drenkow J, Bekiranov S, Strausberg RL, et al. (2002) Large-scale transcriptional activity in chromosomes 21 and 22. Science 296: 916-919.

2. Cawley S, Bekiranov S, Ng HH, Kapranov P, Sekinger EA, et al. (2004) Unbiased mapping of transcription factor binding sites along human chromosomes 21 and 22 points to widespread regulation of noncoding RNAs. Cell 116: 499-509.

3. Wei CL, Wu Q, Vega VB, Chiu KP, Ng P, et al. (2006) A global map of p53 transcription-factor binding sites in the human genome. Cell 124: 207-219.

4. Yang A, Zhu Z, Kapranov P, McKeon F, Church GM, et al. (2006) Relationships between p63 binding, DNA sequence, transcription activity, and biological function in human cells. Mol Cell 24: 593-602.

5. Wu G, Osada M, Guo Z, Fomenkov A, Begum S, et al. (2005) DeltaNp63alpha up-regulates the Hsp70 gene in human cancer. Cancer Res 65: 758-766.

6. Teissier S, Ben Khalifa Y, Mori M, Pautier P, Desaintes C, et al. (2007) A new E6/P63 pathway, together with a strong E7/E2F mitotic pathway, modulates the transcriptome in cervical cancer cells. J Virol 81: 9368-9376.

FIGURE S1


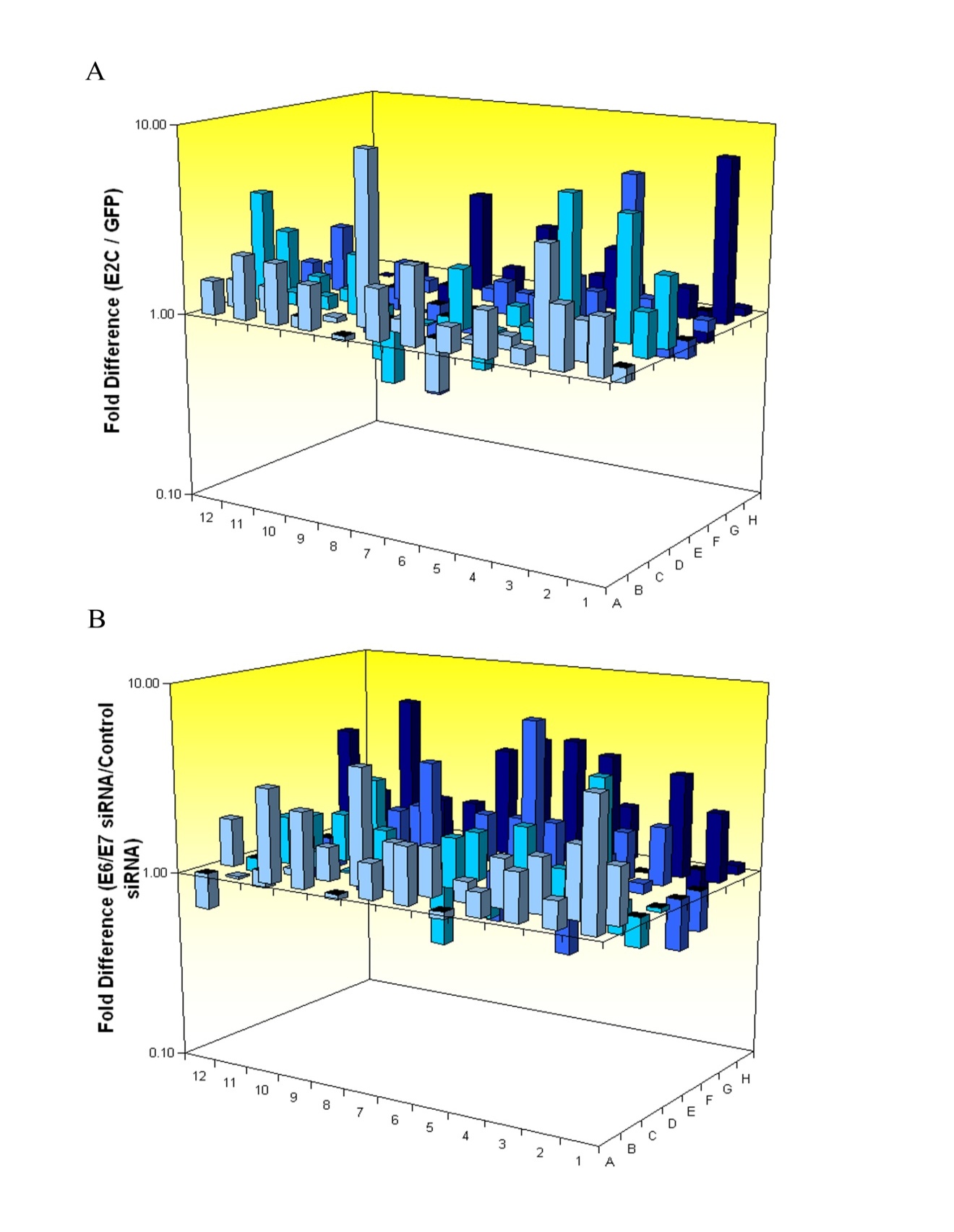


FIGURE S2


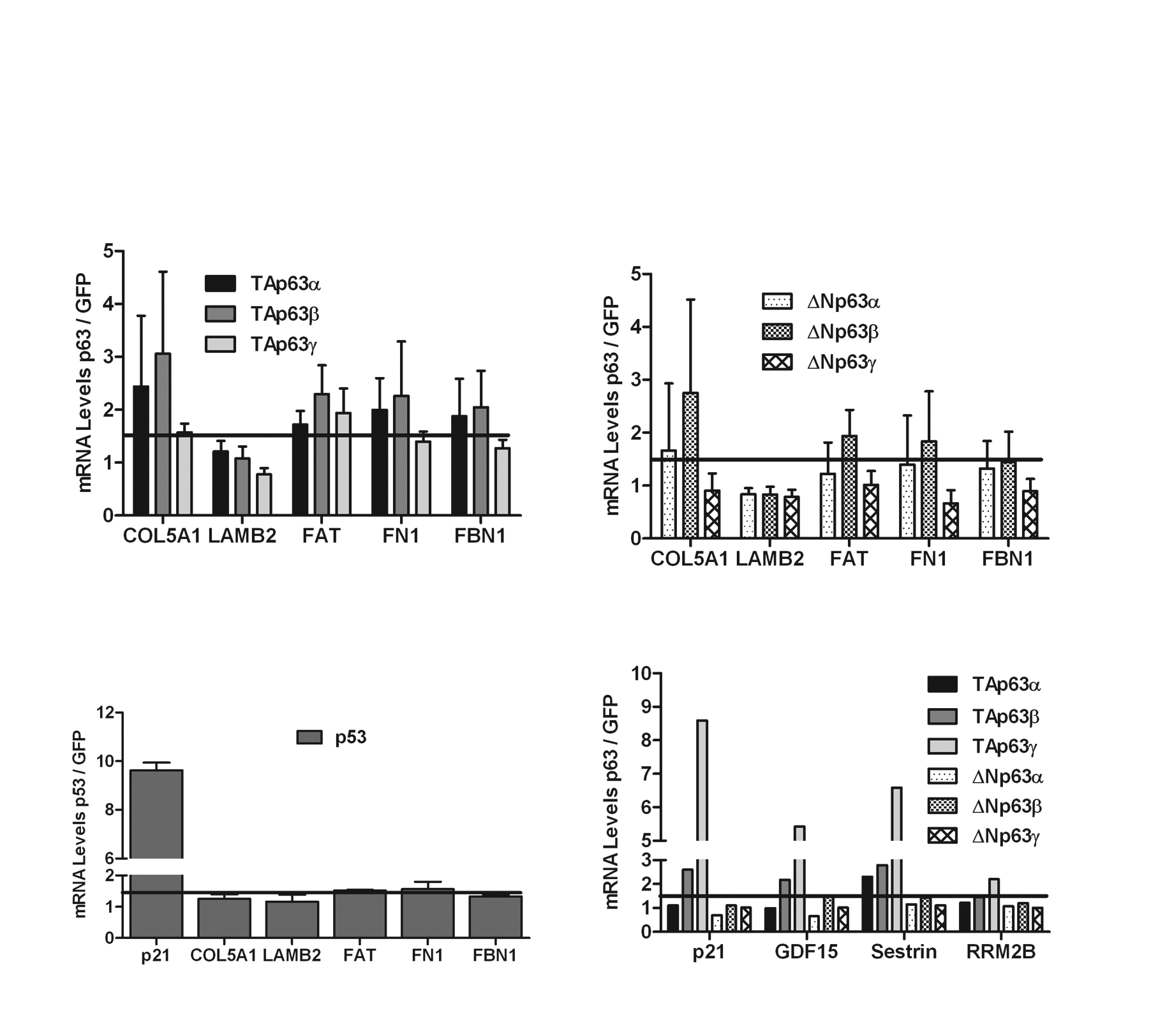


FIGURE S3


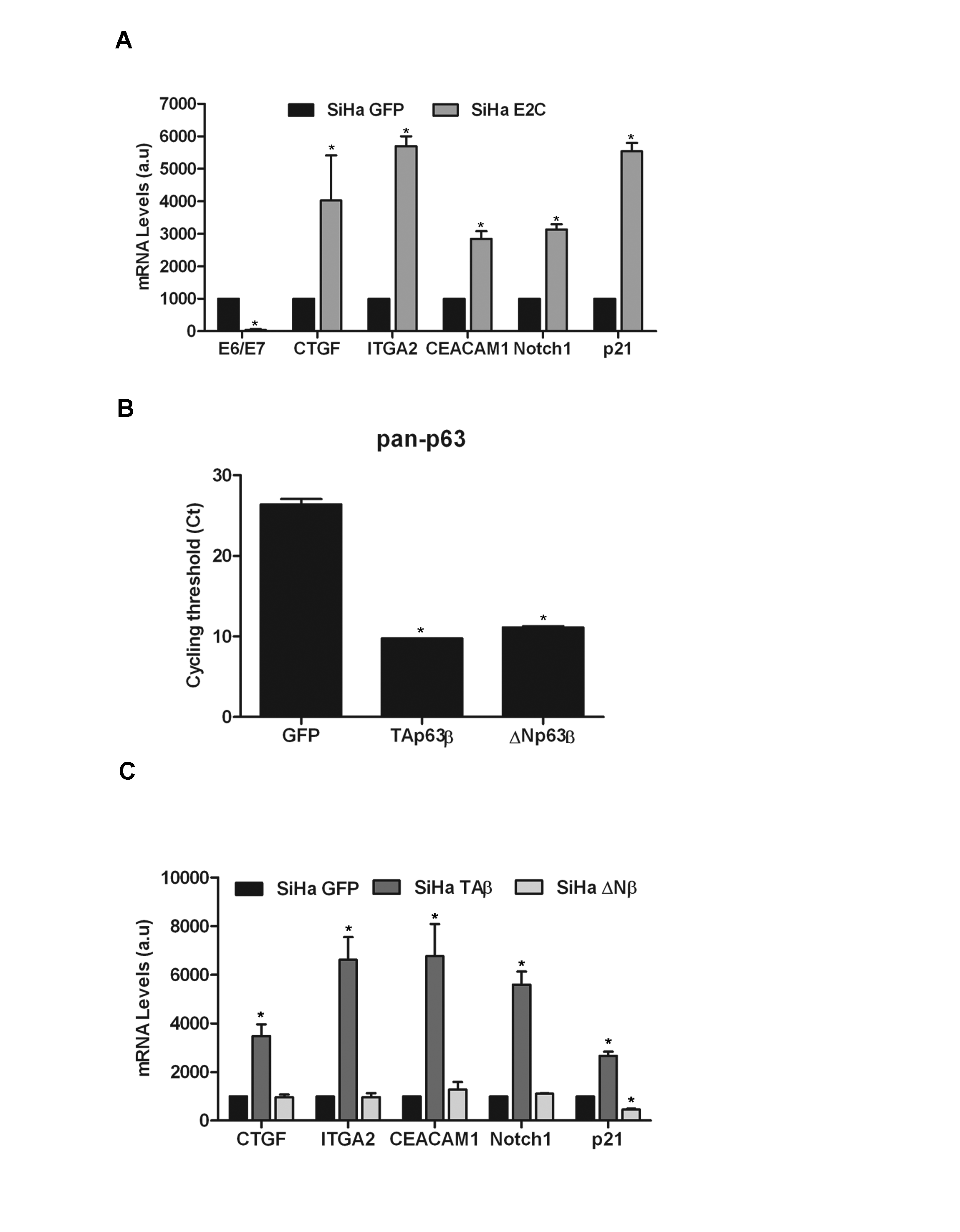


FIGURE S4


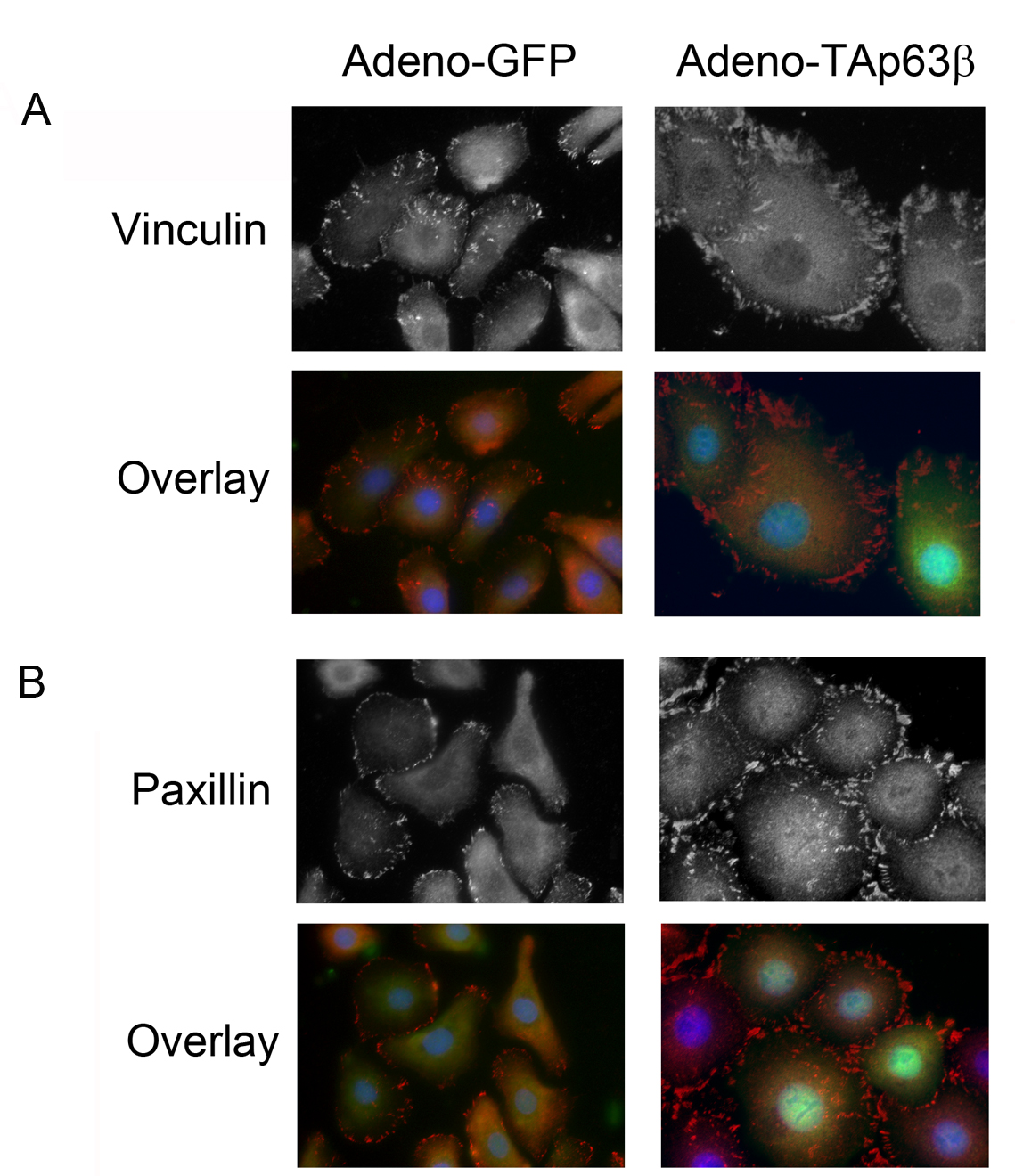


FIGURE S5


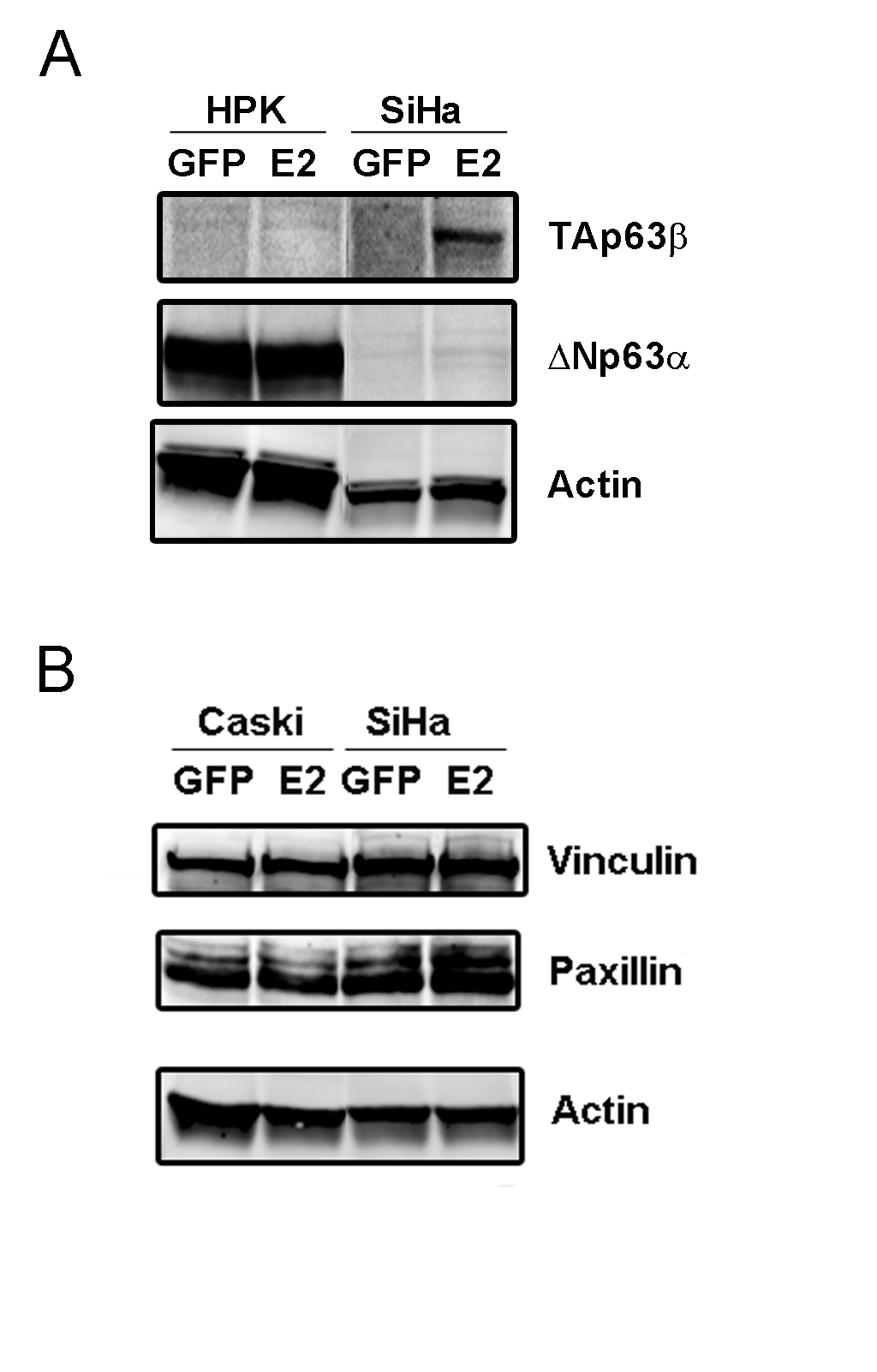


TABLE S3

| **Gene symbol** | **Gene description** | **Function** | **Fold activation** |
| --- | --- | --- | --- |
| ADA | Adenosine deaminase | Regulation of cell-cell adhesion mediated by integrins. | 1.6 |
| ADAM19 | Metallopeptidase domain 19 | Cell adhesion. | 1.8 |
| ADAMTS1 | Metallopeptidase with thrombospondin type 1 motif, 1 | Matrix degrading protein, role in angiogenesis. | 1.5 |
| ANTXR2 | Anthrax toxin receptor 2 | Binds to collagen IV and laminin, extracellular matrix adhesion. | 2.5 |
| CD36 | CD36 molecule (thrombospondin receptor) | Cell adhesion molecule. | 1.6 |
| CEACAM1* | Carcinoembryonic antigen-(CEA) cell adhesion molecule 1 | Mediates cell adhesion. | 2.9 |
| COL4A5 | Collagen, type IV, alpha 5 | Extracellular matrix structural and basement membrane constituent. | 1.9 |
| CTGF* | Connective tissue growth factor | Plays a role in cell-cell and cell-matrix adhesion and migration. | 2.1 |
| DOCK4 | Dedicator of cytokinesis 4 | GTPase activator for adherent junctions between cells. | 1.8 |
| EMP1 | Epithelial Membrane 1 | Adhesion molecule? | 1,7 |
| ETV5 | Ets variant 5 | Transcription factor. | 2.2 |
| FAT1 | FAT tumor suppressor homolog 1 | Member of the cadherin family. | 2.1 |
| FLG | Filaggrin | Key protein facilitating epidermal differentiation and maintaining barrier function. | 3.8 |
| HAS2 | Hyaluronan synthase 2 | Constituent of the ECM. Actively produced during wound healing. | 1.8 |

| HAS3 | Hyaluronan synthase 3 | p63 target, constituent of the ECM. Increased during wound healing. | 1.9 |
| --- | --- | --- | --- |
| HBEGF | Heparin-binding EGF-like growth factor | Involved in migration and cell adhesion. | 1.8 |
| HSPG2 | Heparan sulfate proteoglycan 2 | Major component of basement membrane. | 1.9 |
| ITGA2* | Integrin, alpha 2 | Involved in cell adhesion. | 1.7 |
| ITGA5 | Integrin, alpha 5 | Involved in cell adhesion. | 1.7 |
| IVL | Involucrin | Component of the keratinocytes crosslinked envelope. | 1.5 |
| LAMB3 | Laminin, beta 3 | Laminin belongs to a family of basement membrane proteins. | 1.6 |
| MMP14 | Matrix metallopeptidase 14 (membrane-inserted) | Involved in the breakdown of extracellular matrix. | 2 |
| MMP28 | Matrix metallopeptidase 28 | Involved in the breakdown of extracellular matrix. | 1.6 |
| NID1 | Nidogen 1 | Role in cell interactions with the extracellular matrix. | 2.6 |
| NOTCH1* | Notch homolog 1, translocation-associated | Promotes keratinocyte differentiation. | 1.8 |
| SERPINB5 | Serpin peptidase inhibitor 5 | Inhibition of motility, invasion and metastasis. | 1.5 |
| SLAMF7 | Slam family member 7 | Adhesion of myeloma cell to bone marrow. | 2 |
| TIMP1 | TIMP metallopeptidase inhibitor 1 | Natural inhibitors of the matrix metalloproteinases (MMPs). | 1.6 |
